# International Federation of Clinical Chemistry (IFCC): Scientific Division, Committee on Enzymes. IFCC methods for the measurement of catalytic concentration of enzymes. Part 7. IFCC method for creatine kinase (ATP: creatine (N-phosphotransferase, EC 2.7.3.2). IFCC Recommendation

**DOI:** 10.1155/S1463924690000049

**Published:** 1990

**Authors:** M. Hørder, R. C. Elser, W. Gerhardt, M. Mathieu, E. J. Sampson

**Affiliations:** 1 Odense University Hospital Department of Clinical Chemistry Odense DK-5000 Denmark


Journal of Automatic Chemistry Vol. 12, No. 1 (January–February 1990), pp. 22–40

### International Federation of Clinical Chemistry (IFCC): Scientific Division, Committee on Enzymes†

### *IFCC methods for the measurement of catalytic concentration of enzymes*

### Part 7. IFCC method for creatine kinase (ATP: creatine *N*-phosphotransferase, EC 2.7.3.2). IFCC Recommendation

**Prepared by M. Hørder, R. C. Elser, W. Gerhardt, M. Mathieu and E. J. Sampson**

*This paper forms part of a series of recommendations on measurements of catalytic concentrations of enzymes. Others deal with:*

*Part 1. General conditions [1].*
*(Approved 1979.)*
*Part 2. Method for aspartate aminotransferase [2].*
*(Approved 1985.)*
*Part 3. Method for alanine aminotransferase [3].*
*(Approved 1985.)*
*Part 4. Method for γ-glutamyltransferase [4].*
*Part 5. Method for alkaline phosphatase [5].*
*Part 6. Reference materials for enzyme measurements.*
*(Stage 1, draft 1989 – copy available from Committee Chairman.)*

### 1. Introduction

The principles applied in the selection of the conditions of measurement are those stated in previous publications by this expert panel [1]. Human serum and tissue extracts have been used as the sources of enzymes. The final concentrations of substrates, auxiliary and indicator enzymes have been selected on the basis of experimental evidence and data in the literature dealing with the three dimeric forms of creatine kinase in serum. The method is also suited for the determination of creatine kinase variants which may be present in serum.

### 2. Principle

Creatine kinase (ATP: creatine *N*-phosphotransferase, EC 2.7.3.2, CK) catalyses the reversible *N*- phosphorylation of creatine by the $Mg^{2+}$-ATP complex [6, 7]. Clear distinctions should be made between cytosolic and mitochondrial forms, as well as between tissue and serum forms. This document is primarily concerned with the cytosolic enzymes, although information relating to other forms is given.

The dimeric molecule consists of two elongated polypeptide subunits termed 'B' and 'M'. The two subunits are very similar. Each has a relative molecular mass of 41 300–43 000 [8, 9] and contains one catalytic site and one reactive sulphhydryl group [10].

The nomenclature of the subunits is based on the tissue source of two of the dimeric CK isoenzymes: 'B' for brain and 'M' for muscle. Different cytosolic isoenzymes occur in different tissues [11]. The homologous dimer, CK-MM, predominates in muscle tissue. The hybrid isoenzyme, CK-MB is found in developing skeletal and fully developed cardiac muscle. It also occurs in trace amounts in adult skeletal muscle. The cytosolic CK isoenzymes can also be named on the basis of their electrophoretic mobility towards the anode [12]. Isoenzymes BB, MB and MM of creatine kinase are named CK-1, CK-2 and CK-3, respectively. A fourth CK isoenzyme occurs in mitochondria of myocardium, skeletal muscle, brain and liver tissues, and is thought to be composed of a third type of subunit [13, 14]. Mitochondrial CK isolated from human heart, skeletal muscle, brain, and liver are isolated as macromolecular complexes [15–17].

† Committee Members: R. Rej (US) (Chairman, M. Hørder (DK) M. Mathieu (FR), L. M. Shaw (US) until December 1984, J. H. Strømme (NOR) until December 1984, K. Lorentz (FRG) from 1985.06, and W. Gerhardt (SW) from June 1985.

Optimization experiments were performed in collaboration with the Study Group on Creatine Kinase, Subcommittee on Enzymes of the Committee on Standards, American Association for Clinical Chemistry. Members: R. C. Elser (Chairman), E. J. Sampson, A. R. Henderson, L. G. Morin and D. A. Nealon.

Reprints are available from Dr Mogens Hørder, Odense University Hospital, Department of Clinical Chemistry, DK-5000 Odense C, Denmark; or from Dr Robert Rej, NY State Department of Health, Wadsworth Center for Labs & Research, Empire State Plaza, Albany, New York 12201-0509, USA.

© IFCC 1990

In human serum, CK may be present in various forms:

CK-MM may occur in several forms of which $MM_1$, $MM_2$ and $MM_3$ are the best documented [18].

CK-MB.

CK-BB.

Macro CK Type 1: immunoglobulin-linked isoenzyme forms (predominantly IgG-linked CK-BB [19–21].

Macro CK Type 2: probably a serum form of mitochondrial CK, although conclusive evidence is still lacking [16, 21, 22].

There are no reports of a dimeric mitochondrial CK in serum.

In order to ensure full catalytic activity, the creatine kinase molecule in serum must be reactivated by a reducing sulphhydryl compound.

Different kinetic properties have been found for each of the human creatine kinase isoenzymes, including those of human origin [23–25]. Kinetic data have also been compiled for mitochondrial CK [26]. Therefore, measurement of total creatine kinase in serum requires using an assay system whose reaction conditions are a compromise.

The proposed method for the measurement of the catalytic concentration of creatine kinase in serum is based on the principles proposed by Oliver [27] and later modified by Rosalki [28] and Szasz *et al.* in a series of publications [23, 29–34]. Recommended or standard methods published by professional societies in the United Kingdom [35], FR Germany [36], The Netherlands [37], France [38], Scandinavia [39, 40] and Switzerland [41] are also based on these publications and have also been considered.

The primary reaction (1), catalysed by creatine kinase, and the coupled reactions (2) and (3) are:

$$\text{Creatine phosphate} + \text{ADP} \xrightarrow[\text{EC 2.7.3.2}]{\text{Creatine kinase}} \text{Creatine} + \text{ATP} \quad (1)$$

$$\text{ATP} + D\text{-Glucose} \xrightarrow[\text{EC 2.7.1.1}]{\text{Hexokinase}} \text{ADP} + D\text{-Glucose-6-phosphate} \quad (2)$$

$$D\text{-Glucose-6-phosphate} + \text{NADP}^+ \xrightarrow[\text{EC 1.1.1.49}]{\text{Glucose-6-phosphate-dehydrogenase}} D\text{-Glucono-}\delta\text{-lactone-6-phosphate} + \text{NADPH} + \text{H}^+ \quad (3)$$

The equilibrium in (1) favours the formation of creatine and ATP at pH values around 6–7, due to the higher energy of creatine phosphate as compared to that of ATP and the lower $K_m$ values for ADP and creatine phosphate than for ATP and creatine [42]. This primary reaction is coupled through the auxiliary reaction (2), catalysed by hexokinase, to the NADPH forming indicator reaction (3) catalysed by glucose-6-phosphate dehydrogenase.

*Table 1. IFCC conditions for measurement of creatine kinase.*

| | |
|---|---|
| Creatine phosphate | 30 mmol/l |
| Adenosine-5′-diphosphate (ADP) | 2 mmol/l |
| Imidazole | 100 ml/l |
| Ethylenediaminetetraacetic acid (EDTA) | 2 mmol/l |
| $Mg^{2+}$ | 10 mmol/l |
| *N*-acetyl-*L*-cysteine | 20 mmol/l |
| Adenosine-5′-monophosphate (AMP) | 5 mmol/l |
| $P^1$,$P^5$-Di(adenosine-5′-)penta-phosphate | 10 μmol/l |
| *D*-Glucose | 20 mmol/l |
| Nicotinamide adenine dinucleotide phosphate ($NADP^+$) | 2 mmol/l |
| Hexokinase (E.C. 2.7.1.1) from yeast | 50 μkat/l (3000 U/l) |
| Glucose-6-phosphate dehydrogenase (E.C. 1.1.1.49) from yeast | 33 μkat/l (2000 U/l) |
| Reaction temperature | 30·0 ± 0·05 °C |
| pH (30 °C) | 6·60 ± 0·05 |
| Volume fraction of serum | 0·0435 (1:23) |

Note: The concentrations apply to the complete reaction mixture. The catalytic concentrations of hexokinase and *D*-glucose-6-phosphate dehydrogenase must be determined as described in Appendix B.

### 3. IFCC conditions for measurement

The reaction conditions have been chosen on the basis of univariate and multivariate experiments with sera from healthy individuals, sera from patients with acute myocardial infarction, sera from patients with skeletal muscle diseases, and with isolated human isoenzymes [30, 38–40, 43, 44].

These IFCC conditions are optimized reaction conditions, which are defined [1] as those conditions that are most favourable for both the kinetic reactions and the technical aspects of the measurement, i.e. these conditions do not necessarily provide maximum activity.

The reaction is initiated by creatine phosphate. Following an initial lag phase, substrate conversion proceeds linearly with time and amount of enzyme until deceleration occurs due to build-up of inhibiting NADPH and decreased $NADP^+$ concentration (see table 1).

### 4. Instrumentation and equipment

A thermostatted spectrometer suitable for accurate measurement at 339 nm should be used.

The specifications for the equipment (for example, spectral band width, light path, accuracy of thermostats) should meet those of previous recommendations [1]. Instruments must be capable of monitoring the linear portion of the rate of conversion curve and should display both the initial absorbance of the reaction mixture and absorbance versus time during the measurement interval. The temperature of the reaction mixture in the cuvette must be controlled at 30·0 ± 0·05 °C.

All volumetric glassware used for the preparation of reagents and for pipetting must meet US National

Institute of Standards and Technology (NIST) Class A specifications, American Chemical Society Microchemical specifications (tolerance is 0·997 to 1·003) or other national equivalents. pH meters must be calibrated at 30 ± 1·0 °C by use of a standardized reference buffer (for example, NIST) with a pH value within 1 unit of the reaction measurement pH.†

### 5. Reagents

(1) Imidazole ($C_3H_4N_2$), $M_r$ 68·1.

(2) Creatine phosphate, disodium salt, tetrahydrate ($C_4H_8N_3O_5PNa_2{\cdot}4H_2O$), $M_r$ 327·2.

(3) Magnesium acetate ($C_2H_3O_2Mg{\cdot}4H_2O$), $M_r$ 214·5.

(4) *D*-Glucose ($C_6H_{12}O_6$), $M_r$ 180·2.

(5) Ethylenediaminetetraacetic acid, disodium salt, dihydrate [EDTA] $C_{10}H_{14}O_8N_2Na_2{\cdot}2H_2O$), $M_r$ 372·2.

(6) Adenosine-5′-monophosphate, disodium salt, hexahydrate [AMP] ($C_{10}H_{12}N_5O_7PNa_2{\cdot}6H_2O$), $M_r$ 499·2.

(7) Adenosine-5′-diphosphate, monopotassium salt, dihydrate [ADP] ($C_{10}H_{13}N_5O_{10}P_2K{\cdot}2H_2O$), $M_r$ 501·3.

(8) *N*-Acetyl-*L*-cysteine ($C_5H_9NO_3S$), $M_r$ 163·2.

(9) $P^1$,$P^5$-Di(adenosine-5′-)pentaphosphate, trilithium salt ($C_{20}H_{26}N_{10}O_{22}P_5Li_3$, $M_r$ 934·2.

(10) β-Nicotinamide adenine dinucleotide phosphate, disodium salt [NADP] ($C_{21}H_{26}N_7O_{17}P_3Na_2$), $M_r$ 787·4.

(11) Hexokinase (EC 2.7.1.1) from yeast (lyophilized or in glycerol).

(12) Glucose-6-phosphate dehydrogenase (EC 1.1.1.49) from yeast (lyophilized or in glycerol).

(13) Acetic acid ($C_2H_4O_2$), $M_r$ 60·1.

(14) Sodium hydroxide (NaOH), $M_r$ 40·0.

(15) Sodium chloride (NaCl), $M_r$ 58·45.

### 6. Purity of reagents

The assessment of reagent purity is made on the basis of functional (performance of reagent), chemical (analytical evaluation) and instrumental (absorbance or fluorescence characteristics) tests. Further details are given in Appendix B.

To prevent the growth of microorganisms in solutions, sterilized containers should be used. All solutions should be prepared in calibrated flasks with water meeting the following standards [45]:

Electrical resistivity: ≥2·0 × $10^4$ ohm/m at 25 °C.
pH: 6·0–7·0.
Silicates: <0·1 mg/l.

### 7. Preparation of solutions

*No. Reagent solution for measurement of CK catalytic concentration*

(I) Stock solution of imidazole buffer.

(II) Stock solution of buffer–reagent mixture (without *N*-acetyl-*L*-cysteine or enzymes).

(III) Working solution of buffer–reagent–enzyme mixture.

(IV) Working solution of creatine phosphate (initiating reagent).

(V) NaCl, 154 mmol/l (diluting reagent).

*No. Reagent solution for measurement of auxiliary enzymes – see Appendix B*

(VI) Working solution III with *N*-acetyl-*L*-cysteine but without enzymes.

(VII) Glucose-6-phosphate dehydrogenase, stock solution (approximately 4 mkat/l, diluted 101 fold.

(VIII) Solution VII, diluted 101 fold.

(IX) *D*-Glucose-6-phosphate (initiating reagent).

(X) Working solution III with *N*-acetyl-*L*-cysteine and glucose-6-phosphate dehydrogenase but without hexokinase.

(XI) Hexokinase, stock solution (approximately 4 mkat/l), diluted 101 fold.

(XII) Solution XI, diluted 101 fold.

(XIII) Adenosine-5′-triphosphate (initiating reagent).

(XIV) Gluconate-6-phosphate (initiating reagent).

(XV) Reagent III, diluted 5 times with solution VI.

(I) Stock solution of imidazole acetate buffer (imidazole 127·8 mmol/l, magnesium acetate 12·8 mmol/l, and EDTA 2·6 mmol/l, pH 7·3 at room temperature).

Dissolve the following components in approximately 950 ml of deionized or distilled water, which meets the above-mentioned requirements: imidazole 8·70 g; magnesium acetate tetrahydrate 2·74 g; and ethylenediaminetetraacetic acid, disodium dihydrate 968 mg. Adjust the pH to 7·3 at room temperature (20–26 °C) with acetic acid, 1 mol/l. Add water to a final volume of exactly 1 l.

The absorbance of the buffer solution (I) at 339 nm should be less than 0·050 [39].

† NIST Standard Reference Materials, $KH_2PO_4$ (SRM 186-I-c) and $Na_2HPO_4$ (SRM 186-II-c) can be used as a 25 mmol $kg^{-1}$ solution having a pH of 6·851 at 30 °C. The slope of the pH meter can be adjusted with a solution containing $KH_2PO_4$ (SRM 186-I-c) at 8·695 mmol $kg^{-1}$ and $Na_2HPO_4$ (SRM 186-II-c) at 30·43 mmol $kg^{-1}$, having a pH of 7·403 at 30 °C.

(II) Stock solution of buffer–reagent mixture (imidazole buffer 127·8 mmol/l, with EDTA 2·6 mmol/l, magnesium acetate 12·8 mmol/l, ADP 2·6 mmol/l, AMP 6·4 mmol/l, $P^1$,$P^5$-Di(adenosine-5′-)pentaphosphate 12·8 μmol/l, D-glucose 25·6 mmol/l, $NADP^+$ 2·6 mmol/l, pH 7·10 to 7·15 at room temperature).

Transfer 900 ml of stock imidazole acetate buffer solution I to a 1500 ml beaker containing a magnetic stirrer. Dissolve the following components with stirring at room temperature:

Adenosine-5′-diphosphate, monopotassium salt, dihydrate, 1303 mg.

Adenosine-5′-monophosphate, disodium salt, hexahydrate, 3195 mg.

$P^1$,$P^5$-Di(adenosine-5′-)pentaphosphate, trilithium salt, 12 mg.

*D*-glucose, 4613 mg.

$NADP^+$, disodium salt 2012 mg.

Note: Use of adenine nucleotide salt preparations other than those indicated may require different substance amounts due to different $M_r$ values. Use of free acids will cause difficulty in dissolution of the substances and may require the addition of alkali.

Transfer the solution to 1 l volumetric flask and add stock imidazole acetate buffer solution I to a final volume of exactly 1000 ml. Distribute 90 ml aliquots of this stock solution into 100-ml bottles.

(III) Working solution of buffer–reagent–enzyme mixture (imidazole 115·0 mmol/l, EDTA 2·3 mmol/l, magnesium acetate 11·5 mmol/l EDTA 2·3 mmol/l, magnesium acetate 11·5 mmol/l, ADP 2·3 mmol/l, AMP 5·8 mmol/l, $P^1$,$P^5$-Di-[adenosine-5′-]pentaphosphate 11·5 μmol/l, *D*-glucose 23·0 mmol/l, $NADP^+$ 2·3 mmol/l, *N*-acetyl-*L*-cysteine 23·0 mmol/l, hexokinase 57·5 ukat/l (3450 U/l), and glucose-6-phosphate dehydrogenase 38·2 μkat/l (2300 U/l), with a pH of 6·6 at 30 °C).

Bring 90 ml of stock solution II to room temperature. Dissolve 375 mg of *N*-acetyl-*L*-cysteine in solution II. Adjust the pH, if necessary, to 6·6 at 30 °C with acetic acid, 1 mol/l.

Add a volume of hexokinase from yeast containing 5·8 μkat (350 U) measured at 30 °C (see Appendix B), in order to give a catalytic concentration of 57·5 μkat/l (3450 U/l) in solution III. Add a volume of glucose-6-phosphate dehydrogenase from yeast containing 3·8 μkat (230 U) measured at 30 °C (see Appendix B) in order to give a catalytic concentration of 38·0 μkat/l (2300 U/l) in solution III. Add water to a final volume of 100 ml.

The 339 nm absorbance of this solution should be less than 0·200 [39].

(IV) Working solution of creatine phosphate, 345 mmol/l. Dissolve 1129 mg of creatine phosphate, disodium tetrahydrate in deionized water and bring the volume to 10·0 ml with water.
Working solution IV should have an absorbance at 339 nm of less than 0·050 [39].

(V) Solution of sodium chloride, 154 mmol/l.
Dissolve 0·9 g of sodium chloride in 100 ml of water.

### 8. Stability of solutions

Based on studies given in Nealon [39], the stabilities of these solutions are:

Solution I : 3 months at 4 °C.
Solution II : 2 months at −20 °C.
Solution III: 5 days at 4 °C, 24 hours at room temperature, or 3 weeks at −20 °C.
Solution IV: 3 months at 4 °C or 12 months at −20 °C.

### 9. Specimen procurement, stability, transportation and storage

Serum is the preferred specimen. Plasma should not be used.

Collect blood by venipuncture with minimal manipulation and stasis. Avoid haemolysis to minimize interference by erythrocyte adenylate kinase. Use blood-collection tubes that contain no additives. Physical exercise can increase the creatine kinase catalytic concentration in the blood [46].

Blood cells should be separated from serum with 2 hours of venipuncture.

The stability of the CK isoenzymes varies. At pH 7·5, complexes of immunoglobulins with CK-BB are the most stable [16] followed by MM, mitochondrial CK, MB and BB [17]. At pH 8·5, mitochondrial CK is more stable [16] than MM [21]. Isoenzyme stabilities, defined as less than 5% loss under the specified storage conditions [33, 47], are:

CK-MM: room temperature, 48 h; 4 °C, 2 weeks; −20 °C, 1 month [33].
CK-MB: room temperature, 2 h [33]; 4 °C, 5 days [47].
CK-BB: room temperature, 0·5 h [33]; 4 °C, 1 day [47]; −20 °C, 2 days [47]; −80 °C, 4 days [47].

All isoenzymes are stable in the complete reaction mixture for 30 min at room temperature and 10 min at 37 °C.

Loss of catalytic concentration may be caused by (*a*) thermal inactivation; (*b*) oxidation of reactive thiol groups; (*c*) increasing pH due to loss of $CO_2$; and (*d*) irradiation with light [48]. In addition, the temperature of the fluid used to reconstitute lyophilized serum has been reported to affect catalytic concentration [49].

Specimens should be transported and stored at low temperatures in tightly-closed tubes with minimal air space over the serum and should be protected from light. The catalytic concentration stability is apparently pH dependent, being maximal around pH 6·5 to 7·0 and minimal around pH 8·0 to 8·5 [50, 51]. Stability may thus vary somewht for individual sera in conditions of acidosis or alkalosis and with the isoenzyme distribution.

Total CK catalytic concentration in serum from healthy individuals is comprised largely of CK-MM with 1–3% of CK-MB [52]. In patients with acute myocardial infarction, CK-MB may be increased considerably and is, therefore, of diagnostic usefulness [53]. CK-MB may be elevated in patients with skeletal muscle damage due to excessive exercise, intramuscular injections, surgical operations, multiple trauma, cerebral arterial embolism, convulsions, and malignant hyperthermia [54], and in Duchenne muscular dystrophy and similar musculoskeletal diseases [55].

Elevated catalytic concentrations of CK-BB have been observed in patients following surgical resection of the prostate or of the GI tract, caesarean section and craniotomy [56]. CK-BB has also been found in the serum of patients with various types of cancer [57], colonic infarction [58] and brain damage [59, 60].

### 10. Measurement

### *10.1. Measurement conditions*

Wavelength: 339 (±1 nm).

Bandwidth: ±2 nm.

Light path: 10·0 ± 0·01 mm.

Final volume of reaction mixture: 2·30 ml.

Temperature: 30·0 ± 0·05 °C (thermostatted cuvette compartment).

### *10.2. Handling of solutions*

Before pipetting, the temperature of reagent solutions and of specimen must be brought to the calibration temperature of the pipettes. However, use of other temperatures results in a relative error of only 0·000025 for each °C difference from the pipette calibration temperature and for most situations this error is negligible.

During the preincubation period, the solution in the cuvettes must attain a temperature of 30·0 ± 0·05 °C before initiating the reaction.

### *10.3. Subprocedures that constitute one measurement*

The overall reaction (A), catalysed by creatine kinase and other enzymes, particularly adenylate kinase (EC 2.7.4.3), the sample blank reaction (B) and the reagent blank reaction (C) comprise the individual measurements for rate of conversion. Reaction (C) does not enter directly into the calculation of results (see section 10·5), see table 2.

*Table 2. Composition of the reaction mixtures for determinations of the rate of conversions required for creatine kinase rate measurements.*

| Kind of reaction | Sample | Reagent mixture | Initiating reagent |
|---|---|---|---|
| (A) Overall | Serum | Solution III | Solution IV |
| (B) Sample blank | Serum | Solution III | Water |
| (C) Reagent blank | Solution V | Solution III | Solution IV |

All measurements are made against a cuvette containing reagent grade water.

*Table 3. Analytical system for measurement of the overall creatine kinase rate of conversion.*

| Pipette into cuvettes: | Volume | Substance or catalytic concentration in final, complete mixture | |
|---|---|---|---|
| Solution III | 2·00 ml | Imidazole | 100 mmol/l |
| | | EDTA | 2 mmol/l |
| | | $Mg^{2+}$ | 10 mmol/l |
| | | ADP | 2 mmol/l |
| | | AMP | 5 mmol/l |
| | | $P^1,P^5$-Di(adenosine-5′-)-pentaphosphate | 10 μmol/l |
| | | *N*-acetyl-*L*-cysteine | 20 mmol/l |
| | | *D*-glucose | 20 mmol/l |
| | | $NADP^+$ | 2 mmol/l |
| | | Hexokinase (3000 U/l) | 50 μkat/l |
| | | Glucose-6-phosphate dehydrogenase (2000 U/l) | 33 μkat/l |
| Serum | 0·100 ml | Volume fraction | 0·0435 (1:23) |

Mix carefully, avoiding the loss of any volume of the mixture. Incubate the reaction mixture at 30 °C and wait a minimum of 300 s for full reactivation of creatine kinase and temperature equilibration. Before the following step, solution IV should be at 30 °C.

| | | | |
|---|---|---|---|
| Solution IV | 0·200 ml | Creatine phosphate | 30 mmol/l |

Mix again and wait 120 s for the end of the lag phase. Monitor the increase in absorbance at 339 nm as a function of time, for at least an additional time of 60 s.

The procedure for the overall reaction of the individual measurement is described in table 3. The other two measurements are performed identically by replacing the sample or starting reagent as described in table 2.

### *10.4. Measurement interval*

A lag phase of up to 120 s may occur. The values of A/s (=*a*) of the overall creatine kinase reaction (A) are constant over a period of at least 60 s following the lag phase for sera with catalytic concentrations of creatine kinase up to 40 μkat/l (2400 U/l), provided that the spectrometer is capable of making accurate absorbance readings up to 2·000 A. If the change of absorbance is greater than 0·01/s the serum sample must be diluted with solution V. However, this will lead to inaccuracy due to nonlinearity of the dilution curve (see Appendix A for details).

*10.5. Corrections for blank reactions*

The rate of the overall reaction (A) is corrected for any sample blank reaction (B) as follows:

$$a \text{ corrected} = a_A - a_B.$$

The subscripts, A and B, indicate the composition of the reaction mixtures referred to in table 2. The corrected value of $a$ is used in the following calculations. It equals the true rate of conversion catalysed by creatine kinase.

The reagent blank rate of conversion, C, does not enter into the calculations, but is used to ascertain the quality of the reagents. Its value should be less than a change in absorbance of 0·0007 per 60 s. If it is higher, the purity of the reagents must be reassessed (see Appendix B).

### 11. Calculation

The catalytic concentration, b, of creatine kinase in the sample is calculated as follows:

$$b = \frac{V}{\varepsilon . 1 . v} \cdot a$$

The molar absorption coefficient, ε, of NADPH (30 °C, 339 nm) = 630 $m^2$ $mol^{-1}$ [61, 62].
The light path length, 1, is 0·01 m (=10 mm).
Let the increase in absorbance per second at 339 nm be $a$, $s^{-1}$.

The total reaction volume, V, is $2{\cdot}3 \times 10^{-3}$ l.

The sample volume, v, is $0{\cdot}1 \times 10^{-3}$ l.

$$b = a\left(\frac{2{\cdot}3 \times 10^{-3}\ s^{-1}\ l}{630 \times 0{\cdot}01 \times 0{\cdot}1 \times 10^{-3}\ m^2\ mol^{-1}\ m\ l}\right)$$

$$= a\left(\frac{2{\cdot}3\ mol\ s^{-1}}{0{\cdot}630\ m^3}\right)$$

$$= a(3{\cdot}651\ mol\ s^{-1}\ m^{-3})$$

$$= a(3{\cdot}651\ kat\ m^{-3})$$

$$= a(3651\ \mu kat\ l^{-1})$$

Note: Calculated for a measuring time of 60 s:
Let the increase in absorbance per 60 s at 339 nm be $A$, $(60\ s)^{-1}$
$b = A\ (3651\ \mu mol\ (60\ s)^{-1}\ l^{-1})$
$= A\ (3651\ U/l)$.

### 12. Analytical variability

Data about inaccuracy are not available because no international reference material has yet been established. Preliminary data regarding imprecision appear in table 4 [38].

*Table 4. Imprecision of CK method.*

| Catalytic concentrations (μkat/l) | Coefficients of variations | |
|---|---|---|
| | Within-day | Day-to-day |
| 0·59 | 0·023 | 0·027 |
| 2·69 | 0·009 | 0·013 |
| 5·81 | 0·008 | 0·010 |

For measurement at 339 nm, with sufficiently sensitive spectrometers, the limit of detectability using 180 s monitoring after the end of the lag phase is 24 ± 8 nkat/l (1·4 ± 0·5 U/l [38].

The analytical sensitivity has been established to be an increase in absorbance at 339 nm of 0·001 per 60 s, but will depend on the instrument used.

### 13. Reference ranges

Reference ranges have not yet been determined for this method. However, reference values are available for many of the national methods shown in table 1 of Appendix A. As an example, with the French national recommendation, reference intervals of 0·30 to 1·50 μkat $l^{-1}$ (18 to 90 U $l^{-1}$) and 0·60 to 3·60 μkat $l^{-1}$ (36 to 216 U $l^{-1}$) were found for women (aged 10–45 years) and men (aged 10–60 years), respectively [38].

### References

1. Bowers, Jr, G. N., Bergmeyer, H. U. Hørder, M. and Moss, D. W., Approved recommendations on IFCC methods for the measurement of catalytic concentrations of enzymes, Part I. General considerations concerning the determination of the catalytic concentration of an enzyme in the blood serum or plasma of man. *Clinica Chimica Acta*, **98** (1979), 163. *Journal of Clinical Chemistry and Clinical Biochemistry*, **18** (1980), 89.
2. Bergmeyer, H. U., Hørder, M. and Rej, R., Approved recommendation (1985) on IFCC methods for the measurement of catalytic concentration of enzymes. Part 2. IFCC method for aspartate aminotransferase (L-aspartate: 2-oxoglutarate aminotransferase, EC 2.6.1.1). *Journal of Clinical Chemistry and Clinical Biochemistry*, **24** (1986), 497.
3. Bergmeyer, H. U., Hørder, M. and Rej, R., Approved recommendation (1985) on IFCC methods for the measurement of catalytic concentrations of enzymes. Part 3. IFCC method for alanine aminotransferase (*L*-alanine: 2-oxoglutarate aminotransferase, EC 2.6.1.2). *Journal of Clinical Chemistry and Clinical Biochemistry*, **24** (1986), 481.
4. Shaw, L. M., Stromme, J. H., London, J. L. and Theodorsen, L., IFCC methods for measurement of catalytic concentration of enzymes. Part 4. IFCC method for γ-glutamyltransferase [(γ-glutamyl)-peptide: amino acid γ-glutamyl-transferase, EC 2.3.2.2]. *Journal of Clinical Chemistry and Clinical Biochemistry*, **21** (1983), 633–646, *Clinica Chimica Acta*, **135** (1983), 315.
5. Tietz, N. W., Rinker, A. D. and Shaw, L. M., IFCC methods for the measurement of catalytic concentration of enzymes. Part 5. IFCC method for alkaline phosphatase (orthophosphoric-monoester phosphohydrolase, alkaline optimum, EC 3.1.3.1). *Journal of Clinical Chemistry and*

*Clinical Biochemistry,* **21** (1983), 731–748, *Clinica Chimica Acta,* **135** (1983), 339.

6. Morrison, J. F. and James, E., The mechanism of the reaction catalysed by adenosine triphosphate-creatine phosphotransferase. *Biochemistry Journal,* **97** (1965), 37.
7. Bessman, S. P. and Carpenter, C. L., The creatine–creatine phosphate shuttle. In *Annual Review of Biochemistry,* **54** (1985), (Ed. C. C. Richardson). (Annual Reviews Inc; Palo Alto, CA), 831.
8. Perryman, M. B., Strauss, A. W. and Buettner, T. L., Molecular heterogeneity of creatine kinase isoenzymes. *Biochimica Biophysica Acta,* **747** (1983), 284.
9. Yue, R. H., Palmieri, R. H., Olson, O. E. and Kuby, S. A., Studies on adenosine triphosphate transphosphorylases V. Studies on the polypeptide chains of the crystalline adenosine triphosphate-creatine transphosphorylase from rabbit skeletal muscle. *Biochemistry,* **6** (1967), 3204.
10. Watts, D. C., Creatine kinase (adenosine-5′-triphosphate-creatine phosphotransferase). In *The Enzymes,* Volume 8 (Ed. Boyer, P. D.), (Academic Press, New York/London, 1973), 383.
11. Urdal, P., Urdal, K. and Stromme, J. H., Cytoplasmic creatine kinase isoenzymes quantitated in tissue specimens obtained at surgery. *Clinical Chemistry,* **29** (1983), 310.
12. IUPAC-IUB Commission on Biochemical Nomenclature (CBN), Nomenclature of multiple forms of enzymes. Recommendations (1976). *Journal of Biological Chemistry,* **252** (1977), 5939–5941, *Clinical Chemistry,* **23** (1977), 2163.
13. Blum, H. E., Weber, B., Deus, B. and Gerok, W., The mitochondrial creatine kinase isoenzyme from human heart muscle. In *Creatine Kinase Isoenzymes, Pathophysiology and Clinical Application* (Ed. Lang, H.), (Springer-Verlag, New York, 1981), 19.
14. Grace, A. M., Perryman, M. B. and Roberts, R., Purification and characterization of human mitochondrial creatine kinase, a single enzyme form. *Journal of Biological Chemistry,* **258** (1983), 15346.
15. Wevers, R. A., Reutelingsperger, C. P. M., Dam, B. and Soons, J. B. J., Mitochondrial creatine kinase (EC 2.7.3.2) in the brain. *Clinica Chimica Acta,* **119** (1982), 209.
16. Stein, W., Bohner, J., Steinhart, R. and Eggstein, M., Macro creatine kinase: Determination and differentiation of two types by their activation energies. *Clinical Chemistry,* **28** (1982), 19.
17. Roberts, R. and Grace, A. M., Purification of mitochondrial creatine kinase. Biochemical and immunological characterization. *Journal of Biological Chemistry,* **255** (1980), 2870.
18. Wevers, R. A., Hagelauer, U., Stein, W., Bohner, J., Faust, U., van Landeghem, A. A. J. and Soons, J. B. J., Indices for the age of the creatine kinase M-chain in the blood. *Clinica Chimica Acta,* **148** (1985), 197.
19. Urdal, P. and Landaas, S., Macro creatine kinase BB in serum, and some date on its prevalence. *Clinical Chemistry,* **25** (1979), 461.
20. Stein, W., Bohner, J., Krais, J., Muller, M., Steinhart, R. and Eggstein, M., Macro creatine kinase BB: Evidence for specific binding between creatine kinase BB and immunoglobulin G. *Journal of Clinical Chemistry and Clinical Biochemistry,* **19** (1981). 925.
21. Stein, W., Bohner, J. and Eggstein, M., Creatine kinase variants: Report on the workshop conference of the German Society for Clinical Chemistry held on September 19 to 21, 1982 in Tubingen, FRG. *Journal of Clinical Chemistry and Clinical Biochemistry,* **21** (1983), 859.
22. Kanemitsu, F., Kawanishi, I. and Mizushima, J., The origin of a cathode-migrating creatine kinase found in serum from a cancer patient. *Clinica Chimica Acta,* **122** (1982), 377.
23. Szasz, G. and Gruber, W., Creatine kinase in serum: 4. Differences in substrate affinity among the isoenzymes. *Clinical Chemistry,* **24** (1978), 245.
24. Kenyon, G. L. and Reed, G. H., Creatine kinase: Structure-activity relationships. *Advances in Enzymology,* **54** (1983), 367.
25. Wong, P. C. P. and Smith, A. F., Biochemical differences between the MB and MM isoenzymes of creatine kinase. *Clinica Chimica Acta,* **68** (1976), 147.
26. Kanemitsu, F., Kawanishi, I. and Mizushima, J., Characteristics of mitochondrial creatine kinases from normal human heart and liver tissues. *Clinica Chimica Acta,* **119** (1982), 307.
27. Oliver, I. T., A spectrophotometric method for the determination of creatine phosphokinase and myokinase. *Biochemistry Journal,* **61** (1955), 116.
28. Rosalki, S. B., An improved procedure for serum creatine phosphokinase determination. *Journal of Laboratory and Clinical Medicine,* **69** (1967), 696–705.
29. Szasz, G., Laboratory measurement of creatine kinase activity. In *Proceedings of the 2nd International Symposium on Clinical Enzymology.* (Eds Tietz, N. W., Weinstock, A. and Rogerson, D. O.), (American Association of Clinical Chemistry, Washington DC, 1976), 143.
30. Szasz, G., Gruber, W. and Bernt, E., Creatine kinase in serum: 1. Determination of optimum reaction conditions. *Clinical Chemistry,* **22** (1976), 650.
31. Szasz, G., Gerhardt, W., Gruber, W. and Bernt, E., Creatine kinase in serum: 2. Interference of adenylate kinase with the assay. *Clinical Chemistry,* **22** (1976), 1806.
32. Szasz, G., Gerhardt, W. and Gruber, W., Creatine kinase in serum: 3. Further study of adenylate kinase inhibitors. *Clinical Chemistry,* **23** (1977), 1888.
33. Szasz, G., Gerhardt, W. and Gruber, W., Creatine kinase in serum: 5. Effect of thiols on isoenzyme activity during storage at various temperatures. *Clinical Chemistry,* **24** (1978), 1557.
34. Szasz, G., Waldenstrom, J. and Gruber, W., Creatine kinase in serum: 6. Inhibition by endogenous polyvalent cations, and effect of chelators on the activity and stability of some assay components. *Clinical Chemistry,* **25** (1979), 446.
35. Association of Clinical Biochemists, Working Party on Enzyme Methods of the Scientific and Technical Committee, Proposed methods for determination of some enzymes in blood serum. *Annals of Clinical Biochemistry* (UK), **17** (suppl.), (1980), 1s.
36. Recommendations of the German Society for Clinical Chemistry, Standardization of methods for the estimation of enzyme activities in biological fluids. Standard method for the determination of creatine kinase activity. Revised draft of 1976. *Journal of Clinical Chemistry and Clinical Biochemistry,* **15** (1977), 255.
37. Enzymcommissie van de Netherlandse Vereniging voor Klinische Chemie, Aanbevolen methode voor het meten van katalytische activiteitsconcentraties van enzymen in serum. *Mededelingen,* **5** (1979), 314.
38. Enzymology Commission, Societe Francaise de Biologie Clinique, Recommendations for measuring the catalytic concentration of creatine kinase in human serum (documents C 1976, C′1980). *Annales de Biologie Clinique,* **40** (1982), 138.
39. The Committee on Enzymes of the Scandinavian Society for Clinical Chemistry and Clinical Physiology, Recommended method for the determination of creatine kinase in blood. *Scandinavian Journal of Clinical Laboratory Investigation,* **36** (1976), 711.
40. The Committee on Enzymes of the Scandinavian Society for Clinical Chemistry and Clinical Physiology, Recommended method for the determination of creatine kinase in blood modified by the inclusion of EDTA. *Scandinavian Journal of Clinical Laboratory Investigation,* **39** (1979), 1.

41. Fachkommission der Schweizerischen Gesellschaft für Klinische Chemie, Empfohlene Methode zur Bestimmung von 3 Enzymen im Blutplasma: ASAT-ALAT-CK. *Bull. Schweiz. Ges. Klin. Chem. Suppl.,* **21** (1980), 43.

42. Saks, V. A., Chernousova, G. B., Gukovsky, D. E. *et al.*, Studies of energy transport in heart cells. Mitochondrial isoenzyme of creatine phosphokinase: Kinetic properties and regulatory action of $Mg^{+2}$ ions. *European Journal of Biochemistry,* **57** (1975), 273.

43. Sampson, E. J., Whitner, V. S., Ali, M. and Fast, D. M., Multivariate examination of response surfaces around the reaction conditions for the Scandinavian Society's recommended method for creatine kinase determinations. *Clinical Chemistry,* **30** (1984), 1322.

44. Fast, D. M., Sampson, E. J., Whitner, V. S. and Ali, M., Creatine kinase response surfaces explored by use of factorial experiments and simplex maximization. *Clinical Chemistry,* **29** (1983), 793.

45. NCCLS Approved Standard ASC-3, Specifications for reagent water used in the clinical laboratory. Chairholder: Winstead, M. (National Committee for Clinical Laboratory Standards, Villanova, PA 19085, 1980).

46. LaPorta, M. A., Linde, H. W., Bruce, D. L. and Fitzsimons, E. J., Elevation of creatine phosphokinase in young men after recreational exercise. *Journal of the American Medical Association,* **239** (1978), 2685.

47. Nealon, D. A. and Henderson, A. R., Stability of commonly used thiols and of human creatine kinase isoenzymes during storage at various temperatures in various media. *Clinical Chemistry,* **23** (1977), 816.

48. Morin, L. G., Creatine kinase: Stability, inactivation, reactivation. *Clinical Chemistry,* **23** (1977), 646.

49. Feld, R. D., Brown, L. F., Neri, B. P. and Witte, D. L., Effect of diluent temperature on creatine kinase values found for lyophilized controls and reference sera. *Clinical Chemistry,* **24** (1978), 2039.

50. Nealon, D. A., Pettit, S. M. and Henderson, A. R., Effect of serum pH on storage stability and reaction lag phase of human creatine kinase isoenzymes. *Clinical Chemistry,* **26** (1980), 1165.

51. Bohner, J., Stein, W., Renn, W., Steinhart, R. and Eggstein, M., Stability of macro creatine kinases and creatine kinase isoenzymes compared: Heat inactivation test for determination of thermostable creatine kinases. *Journal of Clinical Chemistry and Clinical Biochemistry,* **19** (1981), 1021.

52. Lott, J. A. and Stang, J. M., Serum enzymes and isoenzymes in the diagnosis and differential diagnosis of myocardial ischemia and necrosis. *Clinical Chemistry,* **26** (1980), 1241.

53. Gerhardt, W., Waldenstrom, J., Hørder, M., Hofvendahl, S., Billstrom, R., Ljungdahl, R., Berning, H. and Bagger, P., Creatine kinase and creatine kinase B-subunit activity in cases of suspected myocardial infarction. *Clinical Chemistry,* **28** (1982), 277.

54. Prellwitz, W., Creatine kinase isoenzymes in direct skeletal muscle damage. In *Creatine Kinase Isoenzymes, Pathophysiology and Clinical Application* (Ed. Lang, H.), (Springer-Verlag, New York, Heidelberg, 1981), 170.

55. Goedde, H. W., Benkmann, H. G., Das, P. K. *et al.*, Activity of creatine kinase isoenzyme MB in serum and red cell acetylcholinesterase variants in patients with Duchenne muscular dystrophy. *Klinische Wochenschrift,* **55** (1977), 215.

56. Tsung, S. H., Several conditions causing elevation of serum CK-MB and CK-BB. *American Journal of Clinical Pathology,* **75** (1981), 711.

57. Silverman, L. M., Dermer, G. B., Zweig, M. H., Van Steirteghem, A. C. and Tokes, Z. A., Creatine kinase BB: A new tumor-associated marker. *Clinical Chemistry,* **25** (1979), 1432.

58. Doran, G. R., Appearance of creatine kinase BB isoenzyme in the serum of a patient suffering from infarction of the colon. *Clinica Chimica Acta,* **92** (1979), 415.

59. Kaste, M., Somer, H. and Kontinnen, A., Brain-type creatine kinase isoenzyme. *Archives of Neurology,* **34** (1977), 142.

60. Becker, M. and Menzel, K., Brain-typical creatine kinase in the serum of newborn infants with perinatal brain damage. *Acta Paediatr. Scand.,* **67** (1978), 177.

61. McComb, R. B., Bond, L. W., Burnett, R. W., Keech, R. C. and Bowers, Jr, G. N., Determination of the molar absorptivity of NADH. *Clinical Chemistry,* **22** (1976), 141.

62. Ziegenhorn, J., Senn, M. and Bucher, T., Molar absorptivities of β-NADH and β-NADPH. *Clinical Chemistry,* **22** (1976), 151.

### Appendix A

### Description of pertinent factors in obtaining optimal conditions for measurements

### 1. Introduction

Efforts to obtain standardized conditions for measuring the catalytic concentration of creatine kinase have led to the appearance of several national recommendations within the last few years (see table A1). They are all rooted in the same methodological principle. The basis for the reaction conditions has been in the publications by Szasz *et al.* during the last decade [8–14]. Re-examination and confirmation of reaction conditions have also been published in the late seventies by groups in North America [15–22]. The Expert Panel on Enzymes of IFCC has followed these developments closely since 1976, when a questionnaire among associate members of the Expert Panel on Enzymes and individual clinical chemists made clear that the IFCC should recommend a method based on these same principles.

Accordingly, the Expert Panel on Enzymes, in close collaboration with some of the members of the national groups represented in table A1, and with members of the working group on creatine kinase of the enzyme subcommittee of the Committee of Standards from the American Association for Clinical Chemistry, have evaluated the conditions of the national methods of table A1.

The following is a review of the various alternatives considered in choosing the final conditions. In addition, validation of the present IFCC method by response surface methodology [23] is presented.

*Table A1. Comparison of recommendations for creatine kinase measurements by National Committees 1976–1982 with this IFCC proposal.*

| | Scandinavian [1, 2] | German [3] | Dutch [4] | Swiss [5] | British [6] | French [7] | IFCC |
|---|---|---|---|---|---|---|---|
| Temperature | 37 °C | 25 °C | 30 °C | 30° (37 °C) | 30 °C | 30 °C | 30 °C |
| pH | 6·5 | 6·7 | 6·7 | 6·6 | 6·6 | 6·6 | 6·6 |
| Imidazole (mmol/l) | 100 | 100 | 100 | 100 | 100 | 100 | 100 |
| $Mg^{2+}$ (as acetate) (mmol/l) | 10 | 10 | 10 | 10 | 10 | 10 | 10 |
| *N*-Acetyl-*L*-cysteine (mmol/l) | 20 | 20 | 20 | 20 | 20 | 20 | 20 |
| ADP (mmol/l) | 2·0 | 2·0 | 2·0 | 2·0 | 2·0 | 2·0 | 2·0 |
| $P^1$,$P^5$-Di(adenosine-5′-)pentaphosphate (μmol/l) | 10 | 10 | 10 | 10 | 10 | 10 | 10 |
| AMP (mmol/l) | 5 | 5 | 5 | 5 | 5 | 5 | 5 |
| *D*-Glucose (mmol/l) | 20 | 20 | 20 | 20 | 20 | 20 | 20 |
| $NADP^+$ (mmol/l) | 2 | 2 | 2 | 2 | 2 | 2 | 2 |
| EDTA (mmol/l) | 2 | — | 2 | 2 | 2 | 2 | 2 |
| Creatine phosphate (mmol/l) | 30 | 30 | 30 | 30 | 30 | 30 | 30 |
| Hexokinase (kU/l)† | 3·5 | 2·5 | 2·5 (25 °C) | 3 | 3 (30 °C) | 3 | 3·0 |
| Glucose-6-phosphate† dehydrogenase (kU/l) | 2·0 | 1·5 | 1·5 (25 °C) | 2·0 | 2 (30 °C) | 2·0 | 2·0 |
| Volume fraction of sample | 0·0435 | 0·0385 | 0·0435 | 0·0333 | 0·0476 | 0·0333 | 0·0435 |
| Preincubation (min) | 5 | 3–5 | 3–5 | 5 | 10 | 5 | 5 |
| Reaction initiated by creatine phosphate | + | + | + | + | + (alternatively serum) | + | + |

† Catalytic concentrations as stated in appropriate references.

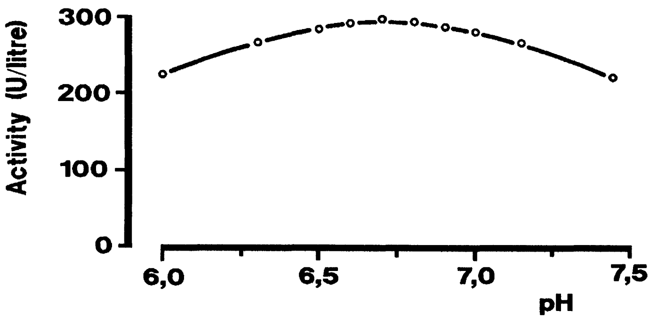

*Figure A1. Dependence of apparent creatine kinase catalytic concentration on pH (100 mmol/l imidazole acetate buffer) at 30 °C [8].*

### 2. Buffer selection

*2.1. pH*

The dependence of creatine kinase activity on pH has been determined by many investigators [1–8]. The activity shows a maximum at pH 6·6, but the difference in activity between pH 6·5 and 6·7 is only 2% (see figure A1, [8]).

*2.2. Buffer type*

The selection of an appropriate buffer for the reaction has been considered by Szasz *et al.* [8] and Morin [15].

The majority of CK investigative work since 1975 has been accomplished using imidazole as the buffer. However, there has been some controversy surrounding the choice of this buffer. Imidazole was difficult to obtain in pure form, and decomposed during storage. This problem has now been solved by the manufacturers and much more stable and pure preparations are available having low absorbance at the wavelength of the measurement [2].

2,2-Bis(hydroxymethyl)-2,2′,2″-nitrilotriethanol (Bis-Tris), due to its chelating ability and buffering capacity at the pH of the assay, has been proposed as an alternate buffer system to imidazole and EDTA [15]. However, studies by Szasz *et al.* [13], by Nealon *et al.* [20], by the SCE [24] and by the CK Study Group of the AACC [25] have shown that there is no distinct superiority of this buffer over imidazole with EDTA.

Studies relating recovery of catalytic concentration to the ionic strength of the buffer show an inverse relationship between catalytic concentration and buffer concentration (see figure A2 and [13]). Buffer anions inhibit creatine kinase. A buffer of 100 mmol/l imidazole containing 2 mmol/l EDTA is therefore a compromise concentration between sufficient buffer capacity and minimal inhibition [8].

### 3. Chelating substance, EDTA

Inclusion of EDTA in the assay has several advantages: it prevents autoxidation of *N*-acetyl-*L*-cysteine and the formation of inhibitors from such oxidation [2, 13], the stability of the CK reagent at 4 °C is increased from less than 24 h to 5 days, and rates of conversion are increased by reversal of the apparent inhibition of CK by endogenous $Ca^{2+}$ [18] and $Fe^{3+}$ [24]. Average increases of apparent CK catalytic concentration in sera from healthy

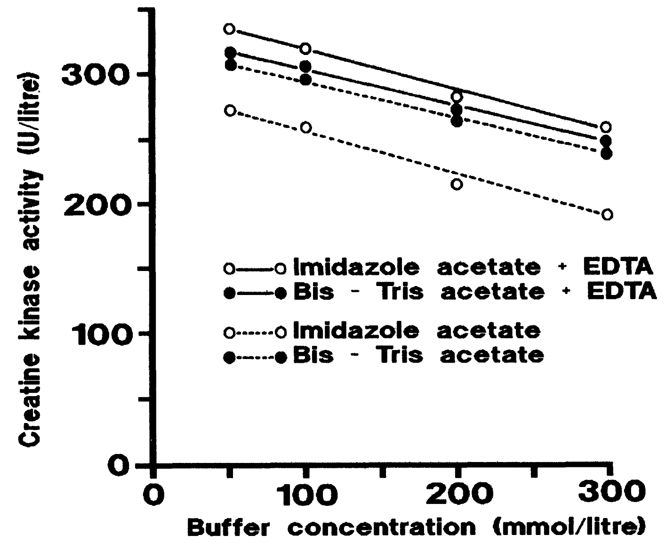

*Figure A2. Dependence of apparent creatine kinase catalytic concentration on buffer concentration at 30 °C [13].*

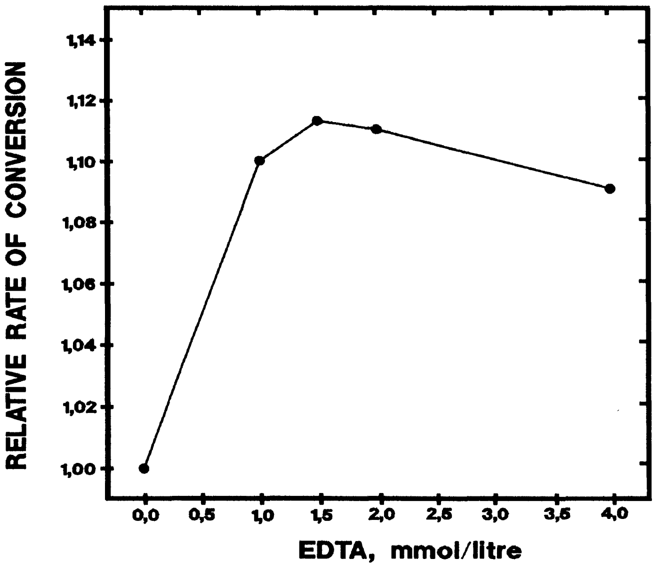

*Figure A3. EDTA influence on creatine kinase catalytic concentration determined in serum, based on data presented in [7].*

individuals and from patients with acute myocardial infarction are from 1·1 to 1·2 times greater measured by an assay at 30 °C containing EDTA, than in an assay, also at 30 °C, without added EDTA [7, 24] (see figure A3, [7]).

### 4. Specimen

### *4.1. Type and volume fraction*

Serum is the preferred specimen. Use of plasma containing heparin, EDTA, or citrate may give rise to unpredictable rates of reactions [7, 24, 26].

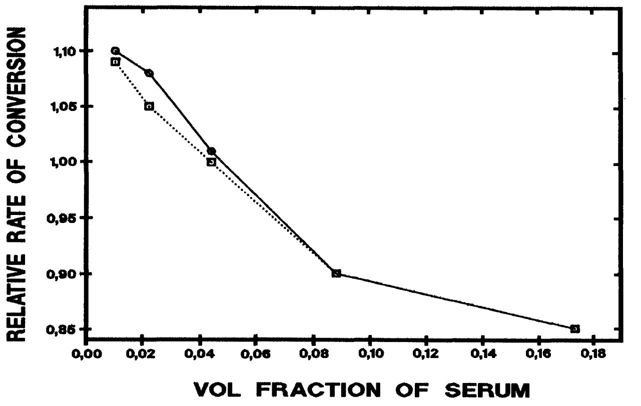

*Figure A4. Effect of serum volume fraction on apparent creatine kinase catalytic concentration [24].*

The volume fraction of the sample is critical. Changes of the volume fraction do not provide proportional changes in the rate of conversion [1]. Therefore, the catalytic concentrations of creatine kinase obtained with this IFCC method are defined specifically at a volume fraction of sample of 0·0435 (see figure A4, [2]).

### *4.2. Reactivation of catalytic activity by* N-*acetyl*-L-*cysteine*

CK in serum is rapidly inactivated. Incubation with a thiol having a high redox potential reactivates CK. This thiol must fulfil several requirements, including rapid and complete reactivation of CK catalytic activity, no precipitation of proteins in the specimen, sufficient solubility in solution and lack of obnoxious odour. Employing freeze drying for incorporation into lyophilized reagent kits is not an absolute requirement for an IFCC method but it is convenient for routine methods [15, 25].

Glutathione was rejected as a reactivator because reactivation is incomplete and requires a longer time. In addition, glutathione reductase in serum causes decreased rates of conversion.

*N*-acetyl-*L*-cysteine has been found to be satisfactory. At a volume fraction of sample of 0·0435 and with 20 mmol/l of *N*-acetyl-*L*-cysteine in the CK reagent, reactivation of CK catalytic activity in serum samples stored for one week at 4 °C is 99% complete [1, 7, 8]. In addition, *N*-acetyl-*L*-cysteine does not cause microprecipitation of sample proteins [1] and is easily soluble at pHs between 6·5 and 6·7 at the required concentration.

Thiols in solution undergo irreversible oxidation. This has two effects: loss of available sulphhydryl groups [12, 16], and formation of potent CK inhibitors [13, 24]. Both processes are accelerated by certain polyvalent cations, and, therefore, are retarded by the presence of chelators. Inclusion of EDTA stabilizes *N*-acetyl-*L*-cysteine in the reagent for 24 h at room temperature and 5 days at 4 °C [2, 13, 24] (see figure A5 and [2]). *N*-acetyl-*L*-cysteine must be of the highest purity to avoid preformed inhibitors [7].

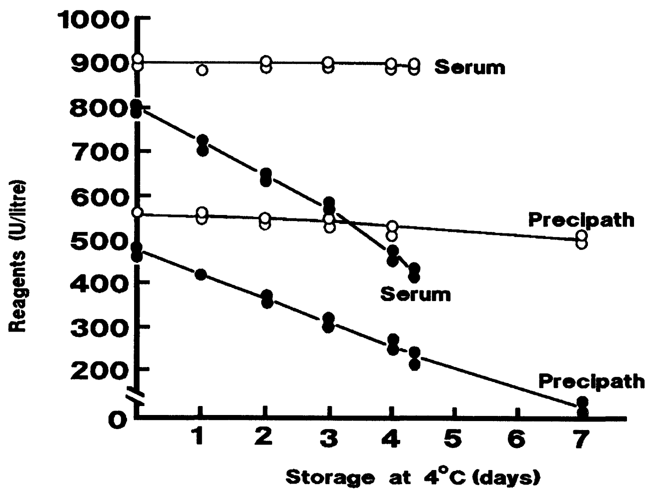

*Figure A5. Stability of CK reagents at 4 °C [24].*

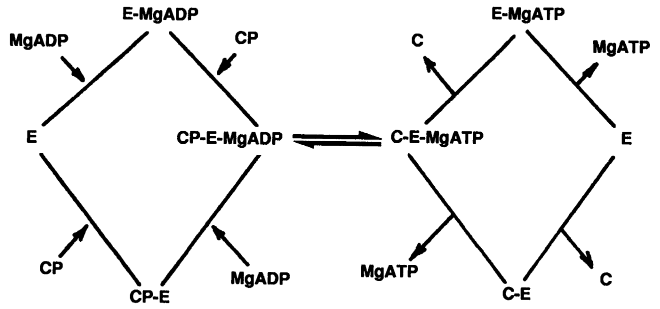

*Figure A6. Reactions involved in the catalytic mechanism of creatine kinase [27].*

### 5. The catalysed reaction

### *5.1. Catalytic mechanisms*

The true substrates of the enzyme should be considered as the magnesium-nucleotide complex, which for this method is Mg-ADP, and free creatine phosphate. The enzyme appears to possess two binding sites on each subunit, one for the nucleotide and one for the guanidino substrate. The site for nucleotide binding presumably involves an arginine group and a lysyl group. It is speculated that a histidyl residue is involved in the guanidino site. Figure A6 has been adopted from Watts [27] and shows the rate equation leading to the formation of the transition complex, and the dissociation of the transition complex into products. It is thought that the process is a rapid equilibrium, random bimolecular mechanism in which either the nucleotide or the guanidino substrate may bind first [27].

Because the nucleotide binds to the active site as the magnesium ion complex, magnesium ion concentration is important in establishing the equilibrium concentration of complexed nucleotide. Wevers *et al.* [28] estimated that the effective equilibrium concentrations of Mg-ADP and creatine phosphate were 1·61 mmol/l and 25·8 mmol/l, respectively, at the nominal reaction conditions.

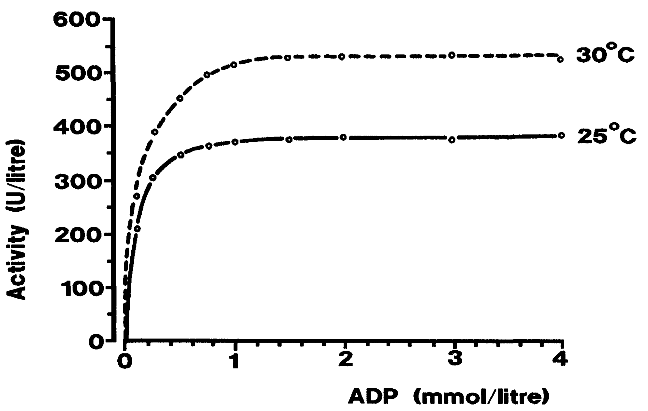

*Figure A7. Dependence of apparent creatine kinase catalytic concentration on ADP concentration [8].*

Small anions can occupy the site normally filled by the gamma-phosphoryl group of magnesium-ATP to form a dead-end complex, which resembles the transition state of the normal enzyme substrate complex. This explains the inhibitory effects of small anions such as nitrate, sulphate, and chloride [27, 29].

### *5.2. Substrate concentrations*

Although the true substrates are Mg-ADP and free creatine phosphate, for the sake of practicality total concentrations are given for ADP and creatine phosphate in the following paragraphs. This convenience is warranted because the influence of imidazole, EDTA, AMP, and other reagent components on the concentration of the respective substrates is unknown.

#### *5.2.1. ADP and magnesium*

Univariate increase of ADP shows a broad plateau with no increase of reaction rates beyond 2 mmol/l [1, 8] (see figure A7, [8]).

Multivariate studies show that simultaneous increases of $Mg^{2+}$ to 15 mmol/l and ADP to 3·5 mmol/l increase CK reaction rates by 6–8% [25, 30, 31] (see Section 8). This change increases the ADP/AMP molar ratio from the current 0·4 to 0·7, and will increase residual adenylate kinase activity [9]. Magnesium is necessary for both creatine kinase and hexokinase activity. The interrelationship between the chelating agents and the substrates, particularly ADP and magnesium and calcium and other divalent cations endogenously present in the sample, is not known at the molecular level. However, the effects of each of these on the rate of conversion have been described [1, 2, 8, 13, 18]. Therefore, magnesium has been selected at a concentration of 10 mmol/l with an EDTA concentration of 2 mmol/l. $K_m$ values for ADP are shown in table A2.

#### *5.2.2. Creatine phosphate*

The $K_m$ values of creatine phosphate for creatine kinase in serum are shown in table A2. Most studies report maximal activity at a broad plateau between 20 mmol/l

*Table A2. Michaelis constants for total ADP and creatine phosphate at 30 °C.*

| Form of Ck | $K_m$ (ADP) | $K_m$ (creatine phosphate) |
|---|---|---|
| $MM_1$ | 0·21 | 2·13 |
| $MM_2$ | 0·24 | 2·87 [28] |
| $MM_3$ | 0·28 | 3·47 |
| MB | 0·17 | 1·2 [11] |
| BB | 0·13 | 0·9 |
| Macro CK type 1 | 0·07 | 1·67 [32] |
| Macro CK type 2 | 0·06 | 0·45 |

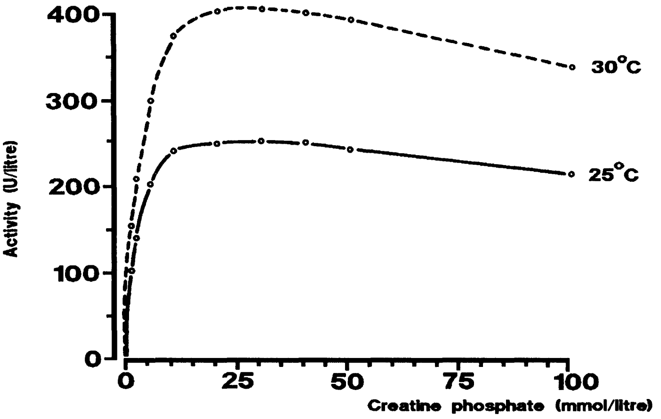

*Figure A8. Dependence of apparent creatine kinase catalytic concentration on creatine phosphate concentration [8].*

and 40 mmol/l. 30 mmol/l has been selected as optimal in both univariate [1, 7 11] (see figure A8, [8]) and multivariate studies [25, 30, 31] (see also Section 8).

### 6. Competitive reaction: interference by adenylate kinase

Adenylate kinase (EC 2.7.4.3, AK) Catalyses the reversible conversion of ADP to ATP and AMP:

$$2\ ADP \xrightarrow{AK} ATP + AMP$$

Adenylate kinase, also known as myokinase, is a remarkably stable enzyme and is present in the same tissues as creatine kinase. Conditions favouring the increased activity of CK also favour the increased activity of adenylate kinase. The pH optima for both enzymes are within two pH units of each other. The overall measured reaction rate includes catalytic activity attributable to adenylate kinase. Consequently, corrections must be made. This is accomplished by including inhibitors of adenylate kinase in the reaction mixture, and, in addition, compensation for a sample blank rate due to the residual adenylate kinase catalytic activity.

Adenylate kinase may be inhibited by various substances, including AMP [1, 9, 10], $P^1$,$P^5$-Di(adenosine-5′-pentaphosphate [9, 10, 33, 36] and sodium fluoride [10, 34, 37].

Maximal inhibition of adenylate kinase by AMP was found when the AMP/ADP ratio was 10:1 [1, 9, 10]. However, AMP also inhibits creatine kinase [1, 9]. CK inhibition is proportional to the concentration of AMP [31] although the degree of inhibition varies among sera. AMP inhibits adenylate kinase by a mechanism which is thought to involve the formation of a 'dead end' complex, either AMP-enzyme-AMP or ADP-enzyme-AMP [35]. Inhibition of adenylate kinase increases as AMP concentration increases and approximately 7% adenylate kinase catalytic activity remains at a concentration of 10 mmol/l of AMP. Because of the competitive nature of the inhibition, the adenylate kinase inhibition also depends on the ADP concentration. Inhibition of CK by AMP also depends on the AMP/ADP ratio [9, 10]. At a ratio of 5, CK is inhibited by approximately 10%. At a ratio of 2·5, CK is inhibited between 5 and 8%. Because both CK and adenylate kinase are sensitive to the AMP/ADP ratio, its value must be selected so that CK is minimally inhibited and adenylate kinase blanks are acceptably low. At an AMP/ADP ratio of 2·5, sample blank rates are decreased significantly, but the highest values of residual adenylate kinase amounted to 20% of the upper limit of the reference range for creatine kinase catalytic activity [10]. The dilemma of reducing residual adenylate kinase without concomitantly inhibiting CK is reduced by adding a second inhibitor.

Diadenosine polyphosphates have been examined as inhibitors of adenylate kinase. The compound with greatest inhibitory capability is $P^1$,$P^5$-Di(adenosine-5′-)-pentaphosphate [36], a potent inhibitor of erythrocyte, muscle, and purified liver adenylate kinase. The pentaphosphate does not inhibit platelet adenylate kinase nearly as well. A combination of AMP and $P^1$,$P^5$-Di(adenosine-5′-)pentaphosphate has been recommended by Szasz et al. [10] at concentrations of 5 mmol/l and 10 μmol/l, respectively. This results in inhibition of adenylate kinase from erythrocytes and muscle by 97%, from liver by 95% [33] and from platelets by 90% [10].

Fluoride is a non-competitive, adenylate kinase inhibitor with a $K_i$ at 25 °C of 2·5 mmol/l [10]. Neither singly, nor in combination with AMP or with $P^1$,$P^5$-Di(adenosine-5′-)pentaphosphate [10] does fluoride offer any advantage over the selected AMP and $P^1$,$P^5$-Di(adenosine-5′-)pentaphosphate inhibitor combination [9, 10]. Fluoride has two disadvantages: it causes turbidity in the reagent due to precipitation of magnesium fluoride, and the lag-time before achieving maximum inhibition by fluoride is 6 min at 30 °C [10]. There are conflicting data regarding inhibition of CK by sodium fluoride; 8% inhibition at 25 mmol/l [10], no inhibition at 25 mmol/l [34], and less than 3% when used in combination with AMP at 6 mmol/l and 2 mmol/l, respectively [37].

The inhibition potentials of the various components are summarized in table A3.

Adenylate kinase catalytic activity not completely inhibited by AMP and $P^1$,$P^5$-Di(adenosine-5′-)pentaphosphate is measured separately by the sample blank

*Table A3. Fractional residual adenylate kinase catalytic activity in the reagent mixture for measurement of creatine kinase with different inhibitors included. Modified from references (1, 7, 9, 10, 33).*

| Inhibitor | | Source of adenylate kinase: Liver† | Liver‡ | Erythrocytes | Thrombocytes | Muscle |
|---|---|---|---|---|---|---|
| AMP | 5 mmol/l | 0·20 | 0·50 | 0·10 | 0·20 | 0·10 |
| $P^1,P^5$-Di(adenosine-5′-)pentaphosphate | 10 μmol/l | 0·08 | 0·50 | 0·03 | 0·20 | 0·03 |
| AMP + $P^1,P^5$-Di(adenosine-5′-)pentaphosphate | 5 mmol/l + 10 μmol/l | 0·06 | 0·05 | 0·03 | 0·10 | 0·01 |
| NaF | 25 mmol/l | | 0·10 | 0·10 | 0·15 | 0·10 |
| NaF + AMP | 25 mmol/l + 5 mmol/l | | 0·05 | 0·02 | — | 0·02 |
| NaF + $P^1,P^5$-Di(adenosine-5′-) pentaphosphate | 25 mmol/l + 10 μmol/l | | 0·05 | 0·01 | — | 0·01 |

† Liver preparation obtained from Blue Sepharose column [33].
‡ Liver extract clarified by centrifugation [33].

rate of conversion, in which creatine phosphate is omitted from the reaction mixture.

In samples from healthy persons this blank rate is zero or very low. In samples from patients with liver and heart diseases, residual adenylate kinase catalytic activity is significant [38]. In addition, in a reference method any possible side-reaction that may contribute to nonspecificity of the method should be compensated for. Therefore, measurement of the sample blank reaction is part of the IFCC method for creatine kinase.

It has been established [39] that creatine phosphate at concentrations from 10 to 90 mmol/l does not inhibit human adenylate kinase from erythrocytes, liver or skeletal muscle.

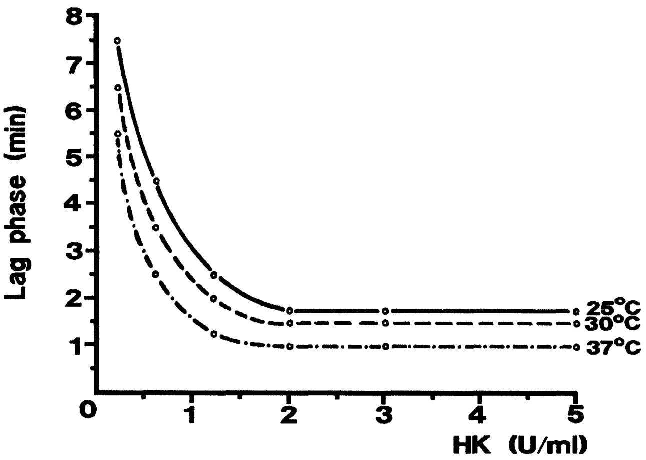

*Figure A9. Lag phase as a function of hexokinase catalytic concentration (U/ml at 25 °C) in an assay mixture containing glucose-6-phosphate dehydrogenase (2 U/ml at 25 °C) [8].*

### 7. Auxiliary and indicator reactions

*7.1. Hexokinase (ATP: D-hexose-6-phosphotransferase, EC 2.7.1.1). Glucose-6-phosphate dehydrogenase (Glucose-6-phosphate: NADP⁺ 1-oxidoreductase, EC 1.1.1.49)*

Hexokinase from baker's yeast is used as the auxiliary enzyme coupling the primary reaction to the indicator reaction. Both the form in which the enzyme is obtained and its final concentration (activity) in the reaction mixture influence the apparent CK activity that is measured. Because of the influence of small anions such as sulphate, nitrate, and chloride on the activity of creatine kinase in the formation of the 'dead end' transition complex (see below), ammonium sulphate suspensions of the auxiliary and indicator enzymes should be avoided. The catalytic concentration of hexokinase influences the duration of the lag phase of the reaction. At hexokinase catalytic concentrations lower than 50 μkat/l (measured at 30 °C in the CK reagent), the lag phase is greater than 120 s (see figure A9, [8]).

The indicator enzyme is glucose-6-phosphate dehydrogenase. Two types of this enzyme are commonly used for analytical purposes: one from yeast, the other from *Leuconostoc mesenteroides*. The coenzyme specificities of the enzymes from these sources are different. The yeast enzyme is specific for $NADP^+$ while the enzyme for *L. mesenteroides* is able to use either $NADP^+$ or $NAD^+$. Because most dehydrogenases present in human sera have specificity for $NAD^+$ and NADH, sera having elevated levels of lactate dehydrogenase may show different CK catalytic concentration when $NAD^+$ is used instead of $NADP^+$. Metabolites capable of acting as substrates for dehydrogenases in the presence of NADH could drive the indicator reaction in the reverse direction and reduce the apparent initial rate of conversion. Results obtained using reagents that included $NADP^+$ and glucose-6-phosphate dehydrogenase from either source were comparable [14]. A reagent formulated to contain glucose-6-phosphate dehydrogenase from the *Leuconostoc* species and $NAD^+$ yielded results that were approximately 6% lower than those obtained using $NADP^+$ [14].

A combination of hexokinase at 50 μkat/l (3000 U/l) and glucose-6-phosphate dehydrogenase at 33 μkat/l (2000 U/l) has been chosen. This is not rate limiting in the measurement of the creatine kinase catalytic concentration below about 40 μkat/l and it ensures a lag phase of less than 120 s (see figure A10, [8]). Under these conditions, the reagent blank rate of conversion will correspond to less than 0·04 μkat/l, provided the reagent specifications described in Appendix B are fulfilled.

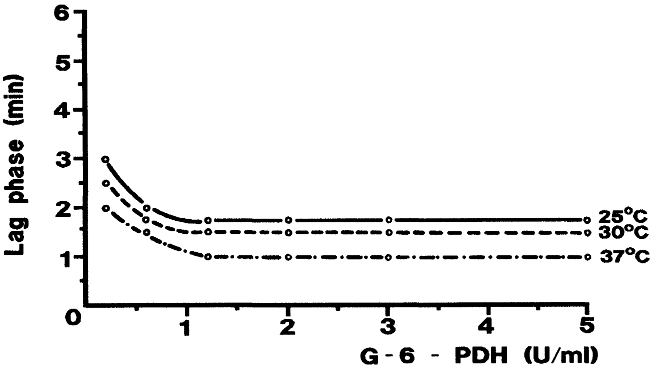

*Figure A10. Lag phase as a function of glucose-6-phosphate dehydrogenase catalytic concentration (U/ml at 25 °C) in an assay mixture containing hexokinase (2 U/ml at 25 °C) [8].*

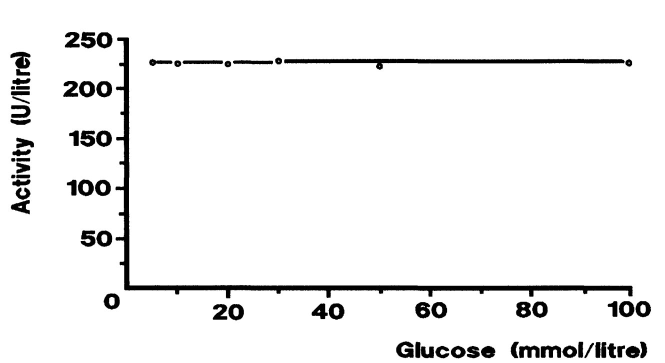

*Figure A11. Effect of glucose concentration on apparent creatine kinase catalytic concentrations [8].*

### *7.2.* D-*Glucose*

Rates of conversion are independent of the glucose concentration in the range of 10 mmol/l to 100 mmol/l. 20 mmol/l provides full catalytic activity with no side-reactions [1, 7, 8] (see figure A11, [8]).

### *7.3. NADP*$^+$

The proportionality between CK catalytic concentration and the rate of conversion also depends on the NADPH $+H^+/NADP^+$ molar ratio. NADPH is a competitive inhibitor of glucose-6-phosphate dehydrogenase [8]. An initial concentration of $NADP^+$ of 2 mmol/l, i.e. about 20 times the average $K_m$ of glucose-6-phosphate dehydrogenase for $NADP^+$ at 37 °C, will sustain a constant rate until NADPH formation has increased the NADPH $+H^+/NADP^+$ molar ratio to about 10. At a volume fraction of sample of 0·0435 this corresponds to sustaining a constant rate of conversion for apparent CK catalytic concentration of 10 μkat/l for 10 min. Rates monitored 120 to 180 s after the start will be linear with a catalytic concentration of CK up to at least 40 μkat/l (2400 U/l) [7, 8].

### 8. Evaluation of the selected conditions by multivariate analysis

The IFCC conditions for the measurement of CK have also been confirmed by multivariate analysis and by response surface analysis. This technique requires simultaneous variation of component concentrations with subsequent computer analysis of the responses obtained at the defined reaction conditions. Results of the computer analysis may be displayed in multidimensional space (usually two dimensions) by plotting the concentration of one variable versus the concentration of a second variable and visualizing the response in terms of iso-response contours. Reaction conditions are interpreted by plateaus, peaks, or valleys in the topography of the response surface [23]. These experiments have been performed by members of the study group for CK of AACC [25, 30, 31].

Reaction surfaces were examined around five variables (imidazole, ADP, creatine phosphate, magnesium, and pH) in the 'Scandinavian' method of table A1 for determining creatine kinase catalytic concentration [30]. Factorial experimentation (five level, five factor) was conducted at reaction temperatures of 30 and 37 °C. Theoretical response surfaces were computed by fitting a quadratic polynomial equation to the experimental data by least-squares regression. Essentially no differences were apparent in the theoretical curves among five human serum specimens analysed at each reaction temperature.

Plots (see figure A12, [30]) of the response-surface data showed the following: for pH and imidazole, maximal catalytic concentration was obtained in the region of the proposed IFCC conditions; the response to changes in creatine phosphate concentration was investigated in respect to changes in concentration of ADP, magnesium acetate and hydrogen ion. A relatively broad plateau of catalytic concentration was observed over the concentrations of the studied variables.

The apparent maxima lie close to the conditions given in this document. For magnesium and ADP, creatine kinase catalytic concentration recovery follows a gently increasing contour as the concentrations of both ADP and magnesium are increased.

Maximal creatine kinase catalytic concentration occurred at magnesium, 15 mmol/l, and ADP 3·5 mmol/l, concentrations greater than those selected in the IFCC method. Full details of these studies have been published by Sampson *et al.* [30, 31].

Optimal concentrations of magnesium and ADP are interdependent [15, 40]. Two effects from increasing total ADP and total magnesium concentrations to 3·5 mmol/l and 15 mmol/l at 30 °C, respectively [30], can be found: (*a*) an increase in the creatine kinase catalysed rate of

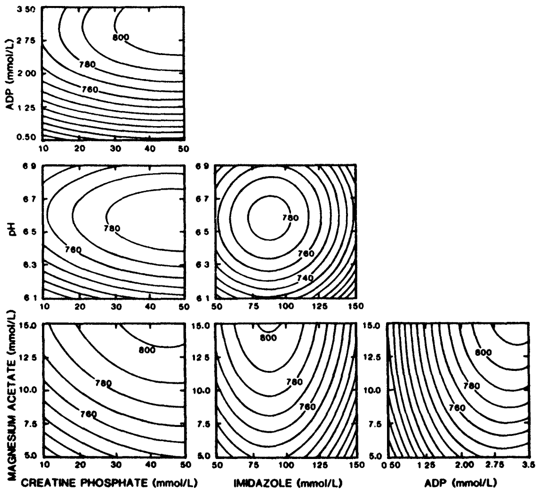

*Figure A12. Optimization of pH, imidazole creatine phosphate, ADP, and magnesium acetate concentration for a human serum using computerized Response Surface Methodology (RSM). The pool was prepared by combining specimens having apparent CK-MB relative catalytic concentrations between 10 and 22%. All other reaction conditions are the same as in this recommended method, except ADP, creatine phosphate and magnesium acetate concentrations which were varied [30].*

conversion of 5–8%; and (*b*) an increase in the nonspecific rate of conversion catalysed by adenylate kinase.

Therefore, the Expert Panel on Enzymes had to consider if these small changes should be included in an IFCC method. This would be necessary if a new method *significantly* improves accuracy, precision, or both. In the absence of primary reference methods for assay of enzymes, accuracy may be considered to be proportional to recovered catalytic concentration. That is to say that methods which result in higher catalytic concentrations for a reference specimen are considered more accurate.

The investment in time and manpower required to validate a new method not only for accuracy but also for transferability, precision, and expected reference values, is substantial. The decision *not* to replace well-accepted conditions as described in this IFCC method [1–8] by slightly changed conditions [30, 31] was therefore made by the Expert Panel on Enzymes. This decision was based on the above criteria and was carefully considered. The 5% to 8% activity increase resulting from the change in total ADP and total magnesium concentrations was felt not to have significant importance to justify a change. Additionally, no improvement in precision was demonstrated.

### References

1. The Committee on Enzymes of the Scandinavian Society for Clinical Chemistry and Clinical Physiology, Recommended method for the determination of creatine kinase in blood. *Scandinavian Journal of Clinical Laboratory Investigation*, **36** (1976), 711.
2. The Committee on Enzymes of the Scandinavian Society for Clinical Chemistry and Clinical Physiology (SCE), Recommended method for the determination of creatine kinase in blood modified by the inclusion of EDTA. *Scandinavian Journal of Clinical Laboratory Investigation*, **39** (1979), 1.
3. Recommendations of the German Society for Clinical Chemistry, Standardization of methods for the estimation of enzyme activities in biological fluids. Standard method for the determination of creatine kinase activity, Revised Draft of 1976. *Journal of Clinical Chemistry and Clinical Biochemistry*, **15** (1977), 255.
4. Enzymcommissie van de Netherlandse Vereniging voor Klinische Chemie, Aanbevolen methode voor het meten van katalytische activiteitsconcentraties van enzymen in serum. *Mededelingen*, **5** (1979), 314.
5. Fachkommission der Schweizerischen Gesellschaft für Klinische Chemie, Empfohlene Methoden zur Bestimmung von 3 Enzymen im Blutplasma: ASAT-ALAT-CK. *Bulletin Schweiz. es. Klin. Chemie* (Supplement), **21** (1980), 43–48.
6. Association of Clinical Biochemists, Working Party on Enzyme Methods of the Scientific and Technical Committee, Proposed methods for determination of some enzymes in blood serum. *Annals of Clinical Biochemistry* (UK), **17** (Supplementum) (1980), 1s.
7. Enzymology Commission, Societe Française de Biologie Clinique, Recommendations for measuring the catalytic concentration of creatine kinase in human serum (documents C 1976, C′ 1980). *Annales de Biologie Clinique*, **40** (1982), 138.
8. Szasz, G., Gruber, W. and Bernt, E., Creatine kinase in serum: 1. Determination of optimum reaction conditions. *Clinical Chemistry*, **22** (1976), 650.
9. Szasz, G., Gerhardt, W., Gruber, W. and Bernt, E., Creatine kinase in serum: 2. Interference of adenylate kinase with the assay. *Clinical Chemistry*, **22** (1976), 1806.
10. Szasz, W., Gerhardt, W. and Gruber, W., Creatine kinase in serum: 3. Further study of adenylate kinase inhibitors. *Clinical Chemistry*, **23** (1977), 1888.
11. Szasz, G. and Gruber, W., Creatine kinase in serum: 4. Differences in substrate affinity among the isoenzymes. *Clinical Chemistry*, **24** (1978), 245.
12. Szasz, G., Gerhardt, W. and Gruber, W., Creatine kinase in serum: 5. Effect of thiols on isoenzyme activity during storage at various temperatures. *Clinical Chemistry*, **24** (1978), 1557.
13. Szasz, G., Waldenstrom, W. and Gruber, W., Creatine kinase in serum: 6. Inhibition by endogenous polyvalent cations and effect of chelator on the activity and stability of some assay components. *Clinical Chemistry*, **25** (1979), 446.
14. Szasz, G., Kinne, E., Colombo, J. P. and Gruber, W., Creatine kinase in serum, VII. Influence of indicating enzyme reaction on apparent creatine kinase activity. *Journal of Clinical Chemistry and Clinical Biochemistry*, **17** (1979), 689.
15. Morin, L. G., Creatine kinase: Re-examination of optimum reaction conditions. *Clinical Chemistry*, **23** (1977), 1569.
16. Nealon, D. A. and Henderson, A. R., Stability of commonly used thiols and of human creatine kinase isoenzymes during storage at various temperatures in various media. *Clinical Chemistry*, **23** (1977), 816.
17. Rollo, J. L., Davis, J. E., Ladenson, J. H., McDonald, J. M. and Bruns, D. E., Effects of β-mercaptoethanol and chelating agents on the stability and activation of creatine kinase in serum. *Clinica Chimica Acta*, **87** (1978), 189.
18. Nealon, D. A. and Henderson, A. R., Effect of cations on the human creatine kinase isoenzymes. *Clinical Chemistry*, **26** (1980), 1137.
19. Nealon, D. A., Pettit, S. M. and Henderson, A. R., Effect of serum pH on storage stability and reaction lag phase of human creatine kinase isoenzymes. *Clinical Chemistry,* **26** (1980), 1165.

20. Nealon, D. A., Pettit, S. M. and Henderson, A. R., [Bis(2-hydroxyethyl)amino]tris(hydroxymethyl)methane is an effective buffer for creatine kinase assays. *Clinical Chemistry,* **26** (1980), 1516.
21. Nealon, D. A., Pettit, S. M. and Henderson, A. R., Activation of human creatine kinase isoenzymes by pH and various sulfhydryl and chelating agents. *Clinical Chemistry,* **27** (1981), 402.
22. Nealon, D. A., Pettit, S. M. and Henderson, A. R., Diluent pH and the stability of the thiol group in monothioglycerol, *N*-acetyl-*L*-cysteine, and 2-mercaptoethanol. *Clinical Chemistry,* **27** (1981), 505.
23. London, J. W., The application of response surface methodology to clinical enzyme assay design. In *Clinical and Analytical Concepts in Enzymology,* Ed. Homburger, H. A. (College of American Pathologists, Skokie, Illinois, 1983), 113.
24. Gerhardt, W., Waldenstrom, J. and Gruber, W., EDTA effect on creatine kinase (CK) and on the SCE reagent. *Scandinavian Journal of Clinical Laboratory Investigation,* **39** (1979), 737.
25. Elser, R., The measurement of creatine kinase. In *Clinical and Analytical Concepts in Enzymology,* Ed. Homburger, H. A. (College of American Pathologists, Skokie, Illinois, 1983), 141.
26. Stromme, J. H. and Theodorsen, L., Heparin interference in the measurement of γ-glutamyl-transferase activity with the Scandinavian and the IFCC recommended method. *Scandanavian Journal of Clinical Laboratory Investigation,* **45** (1985), 437.
27. Watts, D. C., Creatine kinase (adenosine 5′-triphosphate-creatine phosphotransferase). In *The Enzymes,* Boyer, P. D. (Ed.), Volume 8 (Academic Press, New York/London, 1973), 383.
28. Wevers, R. A., Hagelauer, U., Stein, W., Bohner, J., Faust, U., van Landeghem, A. A. J. and Soons, J. B. J., Indices for the age of the creatine kinase M-chain in the blood. *Clinica Chimica Acta,* **148** (1985), 197.
29. Reed, G. H. and McLaughlin, A. C., Structural studies of transition state analog complexes of creatine kinase. *Annals of The New York Academy of Science,* **222** (1973), 118.
30. Sampons, E. J., Whitner, V. S., Ali, M. and Fast, D. M., Multivariate examination of response surfaces around the reaction conditions for the Scandinavian society's recommended method for creatine kinase determinations. *Clinical Chemistry,* **30** (1984), 1322.
31. Fast, D. M., Sampson, E. J., Whitner, V. S. and Ali, M., Creatine kinase response surfaces explored by use of factorial experiments and simplex maximization. *Clinical Chemistry,* **29** (1983), 793.
32. Stein, W., Bohner, J. and Eggstein, M., Creatine kinase variants: Report on the workshop conference of the German Society for Clinical Chemistry held 19–21 September 1982 in Tubingen, FR Germany. *Journal of Clinical Chemistry and Clinical Biochemistry,* **21** (1983), 859.
33. Nealon, D. A., Relative inhibition of human adenylate kinase and creatine kinase isoenzymes by adenosine 5-monophosphate and diadenosine pentaphosphate. *Clinical Chemistry,* **31** (1985), 333.
34. Rosano, T. G., Clayson, K. J. and Strandjord, P. E., Evaluation of adenosine 5′-monophosphate and fluoride as adenylate kinase inhibitors in the creatine kinase assay. *Clinical Chemistry,* **22** (1976), 1078.
35. Rhoads, D. G. and Lowenstein, J. M., Initial velocity and equilibrium kinetics of myokinase. *Journal of Biological Chemistry,* **243** (1968), 3963.
36. Lienhard, G. E. and Secemski, I. I., $P^1$,$P^5$-Di-(adenosine-5′)pentaphosphate, a potent multisubstrate inhibitor of adenylate kinase. *Journal of Biological Chemistry,* **248** (1973), 1121.
37. Meiattini, F., Giannini, G. and Tarli, P., Adenylate kinase inhibition by adenosine 5′-monophosphate and fluoride in the determination of creatine kinase activity. *Clinical Chemistry,* **24** (1978), 498.
38. Gerhardt, W., Waldenstrom, J., Hørder, M., Hofvendahl, S., Billstrom, R., Ljungdahl, R., Berning, H. and Bagger, P., Creatine kinase and creatine kinase B-subunit activity in cases of suspected myocardial infarction. *Clinical Chemistry,* **28** (1982), 277.
39. Nealon, D. A., Creatine phosphate does not inhibit human adenylate kinases. *Clinical Chemistry,* **30** (1984), 1714.
40. Lahet, C., Vialle, A., Maire, I., Parrin, B., Steghens, Y. P. and Mathieu, M., Creatine kinase: reassessment of optimal concentrations for adenosine-5′-diphosphate and magnesium. *Clinical Chemistry,* **32** (1986), 271.

### Appendix B

### Reagent Specifications: Conditions for Measuring the Catalytic Concentration of Auxiliary and Indicator Enzymes and their Contaminants

In the IFCC Method for CK, the auxiliary enzyme is hexokinase (ATP: *D*-hexose-6-phosphotransferase, EC 2.7.1.1, HK) and the indicator enzyme is glucose-6-phosphate dehydrogenase (*D*-glucose-6-phosphate: $NADP^+$ 1-oxidoreductase, EC 1.1.1.49, G-6-P DH). The conditions for measurement of the catalytic concentrations of these two enzymes (temperature, pH, reagent concentrations) correspond to those of this IFCC creatine kinase method.

Both enzymes must be as free as possible from contaminating enzymes, including CK and adenylate kinase, which will cause a significant reagent blank rate. Traces of 6-phosphogluconate dehydrogenase (6-Phospho-*D*-gluconate: $NADP^+$ 2-oxidoreductase (decarboxylating), EC 1.1.1.44) will falsely increase the apparent CK rate due to the production of an additional molecule of NADPH + $H^+$ for each molecule of *D*-glucono-δ-lactone-6-phosphate produced in the indicator reaction.

Hexokinase and glucose-6-phosphate dehydrogenase must be supplied in glycerol, or lyophilized. Standard enzyme suspensions in $(NH_4)_2SO_4$ cause a loss of about 10% in reaction rates [1].

### Measurement of glucose-6-phosphate dehydrogenase catalytic concentration in stock solution

*Principle*

As an example, let the unknown catalytic concentration of glucose-6-phosphate dehydrogenase in the stock solution of enzyme be about 4 mkat/l (240 kU/l 30 °C, this method). This catalytic concentration has to be diluted about 10 000 fold in working solution VI in order to be measured.

*Table B1. Analytical system for measurement of D-glucose-6-phosphate dehydrogenase [EC 1.1.1.49] catalytic activity concentration.*

| Pipette into cuvettes: | Volume | Substance concentration in final, complete mixture | |
|---|---|---|---|
| Solution VIII | 2·00 ml | Imidazole | 100 mmol/l |
| | | EDTA | 2 mmol/l |
| | | $Mg^{2+}$ | 10 mmol/l |
| | | ADP | 2 mmol/l |
| | | AMP | 5 mmol/l |
| | | $P^1$,$P^5$-Di(adenosine-5′-)-pentaphosphate | 10 μmol/l |
| | | *N*-Acetyl-*L*-cysteine | 20 mmol/l |
| | | *D*-Glucose | 20 mmol/l |
| | | $NADP^+$ | 2 mmol/l |
| | | Glucose-6-phosphate dehydrogenase | Approximately 0·4 μkat/l |
| Water | 0·100 ml | Volume fraction | 0·0435 (1:23) |
| Mix carefully. Incubate the reaction mixture at 30 °C and wait a minimum of 300 s for temperature equilibration. | | | |
| Solution IX | 0·200 ml | *D*-Glucose-6-phosphate | 10 mmol/l |
| Mix again and incubate for 120 s. Monitor the increase in absorbance under steady state conditions at 339 nm for at least 60 s. | | | |

(VI) Working solution of buffer-reagent mixture (working solution III prepared without enzymes, pH 6·6 [30 °C]) (see Section 7).

(VII) Dilution of glucose-6-phosphate dehydrogenase, step 1. Pipette 2·00 ml of solution VI into a glass tube. Add 20 μl of the unknown glucose-6-phosphate dehydrogenase stock solution. Mix well.

(VIII) Dilution of glucose-6-phosphate dehydrogenase, step 2. Pipette 2·00 ml of solution VI into a glass tube. Add 20 μl of solution VII. Mix well.

(IX) Initiating reagent (*D*-glucose-6-phosphate, 115 mmol/l). Dissolve 324 mg of *D*-glucose-6-phosphate, monosodium salt ($C_6H_{12}O_9PNa$, $M_r$ 282·2) in 5 ml of solution I. Dilute to 10 ml with water. The final concentration of *D*-glucose-6-phosphate in the assay mixture will be 10 mmol/l.

### Conditions for measurement

The conditions for measurement for glucose-6-phosphate dehydrogenase catalytic concentration are similar to those of the IFCC creatine kinase method, except that hexokinase and creatine phosphate are omitted. Glucose-6-phosphate dehydrogenase is present at a rate limiting catalytic concentration. The reaction is initiated with *D*-glucose-6-phosphate (see table B1).

*Calculation*

The catalytic concentration, b, of glucose-6-phosphate dehydrogenase in the stock solution is calculated as follows:

$$b = \frac{V.\phi}{\varepsilon.l.v} \cdot a.$$

The molar absorption coefficient, ε, of NADPH (30 °C, 339 nm) is 630 $m^2\ mol^{-1}$ [2, 3].

The light path length, l, is 0·01 m (= 10 mm).

Let the increase in absorbance per second at 339 nm be *a* ($s^{-1}$).

The total reaction volume, V, is $2{\cdot}3 \times 10^{-3}$ l.

The 'sample' volume, v, is $2 \times 10^{-3}$ l.

The dilution factor, ϕ, of glucose-6-phosphate dehydrogenase is 101 × 101 = 10 201.

$$b = a\left(\frac{2{\cdot}3 \times 10^{-3} \times 10\,201\ l\ s^{-1}}{630 \times 0{\cdot}01 \times 2 \times 10^{-3}\ m^2\ mol^{-1}\ m\ l}\right)$$

$$= a\left(\frac{23\,462\ mol\ s^{-1}}{12{\cdot}6\ m^3}\right)$$

$$= a(1862\ mol\ m^{-3}\ s^{-1})$$

$$= a(1862\ kat\ m^{-3})$$

$$= a(1862 \times 10^3\ \mu kat\ l^{-1}).$$

Note: Calculated for a measuring time of 60 s:

Let the increase in absorbance per 60 s at 339 nm be *A* $(60\ s)^{-1}$.

$b = A(1862 \times 10^3\ \mu mol\ (60\ s)^{-1}\ l^{-1})$

$= A(1862 \times 10^3\ U/l)$.

Example: Let the increase in absorbance at 339 nm = 0·130 $(60\ s)^{-1}$.
b = 242 kU/l (= 4·034 mkat/l).

### Measurement of hexokinase catalytic concentration in stock solution

*Principle*

As an example, let the unknown catalytic concentration of hexokinase in the stock solution of enzyme be about 4 mkat/l (240 kU/l 30 °C, this method). This catalytic concentration has to be diluted about 10 000 fold in working solution X in order to be measured.

(X) Working solution of buffer–reagent–enzyme mixture (working solution III prepared without hexokinase, pH 6·6 [30 °C]) (see Section 7).

(XI) Dilution of hexokinase, step 1. Pipette 2·00 ml of solution X into a glass tube. Add 20 μl of the unknown hexokinase stock solution. Mix well.

(XII) Dilution of hexokinase, step 2. Pipette 2·00 ml of solution X into a glass tube. Add 20 μl of solution XI. Mix well.

(XIII) Initiating reagent (adenosine-5′-triphosphate [ATP], 115 mmol/l. Dissolve 696 mg of

*Table B2. Analytical system for measurement of hexokinase catalytic concentration.*

| Pipette into cuvettes: | Volume | Substance or catalytic concentration in final, complete mixture | |
|---|---|---|---|
| Solution XII | 2.00 ml | Imidazole | 100 mmol/l |
| | | EDTA | 2 mmol/l |
| | | $Mg^{2+}$ | 10 mmol/l |
| | | ADp | 2 mmol/l |
| | | AMP | 5 mmol/l |
| | | $P^1$,$P^5$-Di(adenosine-5-)-pentaphosphate | 10 µmol/l |
| | | *N*-Acetyl-*L*-cysteine | 20 mmol/l |
| | | *D*-Glucose | 20 mmol/l |
| | | $NADP^+$ | 2 mmol/l |
| | | Hexokinase | Approximately 0·4 µkat/l |
| | | Glucose-6-phosphate dehydrogenase (2000 U/l) | 33 µkat/l |
| Water | 0·100 ml | Volume fraction | 0·0435 (1:23) |

Mix carefully. Incubate the reaction mixture at 30 °C and wait a minimum of 300 s for temperature equilibration.

| | | | |
|---|---|---|---|
| Solution XIII | 0·200 ml | Adenosine-5′-triphosphate | 10 mmol/l |

Mix again and incubate for 120 s. Monitor the increase in absorbance under steady state conditions at 339 nm for 60 s.

adenosine-5′-triphosphate, disodium salt ($C_{10}H_{14}N_5O_{13}P_3Na_2{\cdot}3H_2O$, in 5 ml of solution I (see Section 7). Dilute to 10 ml with water. The final concentration of adenosine-5′-triphosphate (ATP) in the assay mixture will be 10 mmol/l. Freshly prepared solution XIII must be used.

*Conditions for measurement*

The conditions for measurement of hexokinase catalytic concentration are similar to those of the IFCC creatine kinase method, except that creatine phosphate is omitted. Hexokinase is present at a rate limiting catalytic concentration. The reaction is initiated with adenosine-5′-triphoshpate (ATP) (see table B2).

*Calculation*

The catalytic concentration, b, of hexokinase in the stock solution is calculated as follows:

$$b = \frac{V{\cdot}\phi}{\varepsilon.l.v}\cdot a.$$

The molar absorption coefficient, ε, of NADPH (30 °C, 339 nm) is 630 $m^2\ mol^{-1}$ [2, 3].

The light path length, l, is 0·01 m (= 10 mm).

Let the increase in absorbance at 339 nm be $a$ ($s^{-1}$).

The total reaction volume, V, is $2{\cdot}3 \times 10^{-3}$ l.

The 'sample' volume, v, is $2 \times 10^{-3}$ l.

The dilution factor, φ, of hexokinase is 101 × 101 = 10 201.

$$b = a\left(\frac{2{\cdot}3 \times 10^{-3} \times 10\,201\ l\ s^{-1}}{630 \times 0{\cdot}01 \times 2 \times 10^{-3}\ m^2\ mol^{-1}\ m\ l}\right)$$

$$= a\left(\frac{23\,462\ mol\ s^{-1}}{12{\cdot}6\ m^3}\right)$$

$$= a(1862\ mol\ m^{-3}\ s^{-1})$$

$$= a(1862\ kat\ m^{-3})$$

$$= a(1862 \times 10^3\ \mu kat\ l^{-1}).$$

Note: Calculated for a measuring time of 60 s:

Let the increase in absorbance per 60 s at 339 nm be $A$, $(60\ s)^{-1}$.

$b = A(1862 \times 10^3\ \mu mol\ (60\ s)^{-1}\ l^{-1})$

$= A(1862 \times 10^3\ U/l)$.

Example: Let the increase in absorbance at 339 nm = $0{\cdot}130\ (60\ s)^{-1}$
b = 242 kU/l (= 4034 mkat/l).

### Measurement of contaminating 6-phospho-*D*-gluconate: $NADP^+$2-oxidoreductase (decarboxylating), [EC 1.1.1.44] in the IFCC assay for creatine kinase

The presence of 6-phospho-*D*-gluconate dehydrogenase will cause a reagent blank rate due to $NADPH{\cdot}H^+$ production by the following reaction [4]:

$$\text{6-phospho-}D\text{-gluconate} + NADP^+ \xrightarrow[\text{[EC. 1.1.1.44]}]{\text{6-phospho-}D\text{-gluconate dehydrogenase}} \text{ribulose-5-phosphate} + CO_2 + NADPH{\cdot}H^+.$$

The conditions for measurement of 6-phospho-*D*-gluconate dehydrogenase catalytic activity concentration are similar to those of the IFCC creatine kinase method, except that creatine phosphate is omitted. The reaction is initiated with 6-phospho-*D*-gluconate.

(XIV) Initiating reagent (6-phospho-*D*-gluconate, 23 mmol/l). Dissolve 87 mg of 6-phospho-*D*-gluconate, trisodium salt ($C_6H_{10}O_{10}PNa_3{\cdot}2H_2O$, $M_r$ 378·1) in 5 ml of solution I. Fill up to 10 ml with water. The final concentration of 6-phospho-*D*-gluconate in the assay mixture will be 2 mmol/l.

*Conditions for detection of 6-phospho-*D*-gluconate: $NADP^+$ 2-oxidoreductase (decarboxylating)*

The conditions for detection of 6-phospho-*D*-gluconate: $NADP^+$ 2-oxidoreductase catalytic concentration are similar to those of the IFCC creatine kinase method, except that creatine phosphate is omitted. The reaction is initiated with 6-phospho-*D*-gluconate. The concentration of 6-phospho-*D*-gluconate corresponds to that which theoretically might arise from conversion of all $NADP^+$ in the assay mixture. This concentration does not necessarily support a linear rate of conversion, but will detect any significant interference from contaminating 6-phospho-*D*-gluconate dehydrogenase (see table B3).

*Table B3. Analytical system for the detection of 6-phospho-*D*-gluconate: NADP 2-oxidoreductase (decarboxylating) [EC 1.1.1.44].*

| Pipette into cuvettes: | Volume | Substance or catalytic concentration in final, complete mixture | |
|---|---|---|---|
| Solution III | 2·00 ml | Imidazole | 100 mmol/l |
| | | EDTA | 2 mmol/l |
| | | $Mg^{2+}$ | 10 mmol/l |
| | | ADP | 2 mmol/l |
| | | AMP | 5 mmol/l |
| | | $P^1$,$P^5$-Di(adenosine-5′-)-pentaphosphate | 10 µmol/l |
| | | *N*-Acetyl-*L*-cysteine | 20 mmol/l |
| | | *D*-Glucose | 20 mmol/l |
| | | $NADP^+$ | 2 mmol/l |
| | | Hexokinase (3000 U/l) | 50 µkat/l |
| | | Glucose-6-phosphate dehydrogenase (2000 U/l) | 33 µkat/l |
| Water | 0·100 ml | Volume fraction | 0·0435 (1:23) |

Mix carefully. Incubate the reaction mixture at 30 °C and wait a minimum of 300 s for temperature equilibration.

| | | | |
|---|---|---|---|
| Solution XIV | 0.200 ml | 6-Phospho-*D*-gluconate | 2 mmol/l |

Mix again and incubate for 120 s. Monitor the increase of absorbance at 339 nm for at least 60 s.

### Evaluation

If the increase of absorbance at 339 nm exceeds 0·001 per 60 s, this may be due to the presence of contaminating 6-phospho-*D*-gluconate dehydrogenase in either the hexokinase or in the glucose-6-phosphate dehydrogenase stock solution. Consequently, the enzyme preparation in question must be replaced.

### References

1. The Committee on Enzymes of the Scandinavian Society for Clinical Chemistry and Clinical Physiology, Recommended method for the determination of creatine kinase in blood. *Scandinavian Journal of Clinical Laboratory Investigation,* **36** (1976), 711.
2. McComb, R. B., Bond, L. W., Burnett, R. W., Keech, R. C. and Bowers, Jr., G. N., Determination of the molar absorptivity of NADH. *Clinical Chemistry,* **22** (1976), 141.
3. Ziegenhorn, J., Senn, M. and Bucher, T., Molar absorptivities of β-NADH and β-NADPH. *Clinical Chemistry,* **22** (1976), 151.
4. Gerhardt, W., Specific determination of glucose-6-phosphate dehydrogenase in erythrocytes using the LKB 8600: Extraction procedures, stability studies, and reference values calculated per mean erythrocyte. *4th International Symposium on Clinical Enzymology* (Eds Burlina, A. and Galzigna, L.) (Conegliano Veneto, Italy, 1972), 464.

